# Non-Canonical NF-κB Activation and Abnormal B Cell Accumulation in Mice Expressing Ubiquitin Protein Ligase-Inactive c-IAP2

**DOI:** 10.1371/journal.pbio.1000518

**Published:** 2010-10-26

**Authors:** Dietrich B. Conze, Yongge Zhao, Jonathan D. Ashwell

**Affiliations:** Laboratory of Immune Cell Biology, Center for Cancer Research, National Cancer Institute, National Institutes of Health, Bethesda, Maryland, United States of America; St. Jude Children's Research Hospital, United States of America

## Abstract

Chromosomal translocations between loci encoding MALT1 and c-IAP2 are common in MALT lymphomas. The resulting fusion proteins lack the c-IAP2 RING domain, the region responsible for its ubiquitin protein ligase (E3) activity. Ectopic expression of the fusion protein activates the canonical NF-κB signaling cascade, but how it does so is controversial and how it promotes MALT lymphoma is unknown. Considering recent reports implicating c-IAP1 and c-IAP2 E3 activity in repression of non-canonical NF-κB signaling, we asked if the c-IAP2/MALT fusion protein can initiate non-canonical NF-κB activation. Here we show that in addition to canonical activation, the fusion protein stabilizes NIK and activates non-canonical NF-κB. Canonical but not non-canonical activation depended on MALT1 paracaspase activity, and expression of E3-inactive c-IAP2 activated non-canonical NF-κB. Mice in which endogenous c-IAP2 was replaced with an E3-inactive mutant accumulated abnormal B cells with elevated non-canonical NF-κB and had increased numbers of B cells with a marginal zone phenotype, gut-associated lymphoid hyperplasia, and other features of MALT lymphoma. Thus, the c-IAP2/MALT1 fusion protein activates NF-κB by two distinct mechanisms, and loss of c-IAP2 E3 activity in vivo is sufficient to induce abnormalities common to MALT lymphoma.

## Abstract 2

Loss of c-IAP2 ubiquitin ligase activity, which occurs in the lymphoma-causing c-IAP2/MALT1 fusion protein, activates non-canonical NF-κB signaling and results in B cell abnormalities characteristic of MALT lymphoma.

## Author Summary

MALT (mucosal associated lymphoid tissue) lymphomas commonly express a mutant protein that contains a portion of the ubiquitin protein ligase cellular Inhibitor of Apoptosis 2 (c-IAP2) and a portion of the paracaspase MALT1. Expression of this fusion protein activates the anti-apoptotic transcription factor NF-κB, but how it does so and whether or not this activity contributes to lymphomagenesis is not known. Here we identify the mechanisms by which the fusion protein activates NF-κB and show that absence of c-IAP2 ubiquitin protein ligase activity in mice, as is the case in patients that express the fusion protein, results in spontaneous activation of NF-κB and many of the phenotypic cellular features of MALT lymphoma. Our findings demonstrate that c-IAP2 ubiquitin protein ligase activity dampens constitutive NF-κB activity and maintains B cell homeostasis, and provide genetic evidence that the loss of this enzymatic activity in the fusion protein has a major contributing role in MALT lymphomagenesis.

## Introduction

The defining characteristic of the IAP (Inhibitor of Apoptosis) gene family is the presence of one or more baculovirus IAP repeats (BIRs) (reviewed in [1]). These ∼70 amino acid regions mediate protein-protein interactions, and in the context of adjacent sequences are responsible for the association of certain IAP family members with caspases. There are eight mammalian IAPs. Some IAPs also contain a RING motif that confers ubiquitin protein ligase (E3) activity. c-IAP1 and c-IAP2 are such RING-containing proteins that bind caspase-7 or -9 but, unlike XIAP, do not inhibit their enzymatic activities [2]. c-IAP1 and c-IAP2 also bind the adaptor protein TNF Receptor Associated Factor 2 (TRAF2) and are components of the Tumor Necrosis Factor Receptor 1 (TNF-R1) and 2 signaling complexes [3]. Upon TNF-R2 occupancy, c-IAP1, but not c-IAP2, ubiquitinates TRAF2 and the mitogen activated protein (MAP) kinase kinase kinase ASK1, resulting in the proteasomal degradation of all three proteins, cessation of MAPK signaling, and an increased susceptibility to cell death [4]–[6].

An emerging body of evidence has implicated the c-IAPs in regulating the activation of the transcription factor NF-κB. NF-κB can be activated by two distinct signaling mechanisms (reviewed in [7],[8]). The most common (the canonical pathway) depends on IκB kinase (IKK) β-mediated phosphorylation of inhibitory IκB proteins, leading to their ubiquitination and degradation. This frees cytosolic NF-κB heterodimers to translocate to the nucleus and regulate gene transcription. The second activating mechanism (the non-canonical pathway) is downstream of a limited number of receptors, including CD40, lymphotoxin β receptor, and BAFF receptors, and involves the proteolytic removal of carboxy-terminal ankyrin motifs in the NF-κB protein p100 to yield p52 [9],[10]. p52/Rel B-dimers translocate to the nucleus and regulate gene transcription [11]. Processing of p100 to p52 is dependent on the sequential activation of the upstream kinases NIK (NF-κB-inducing kinase) and IKKα [12]–[14]. Transient overexpression of c-IAP2 in cells has been shown to induce the ubiquitination and degradation of IκB, the essential antigen receptor NF-κB signaling intermediate Bcl-10, and NIK [15]–[18]. Overexpression of c-IAP1 induced the ubiquitination and degradation of TRAF2 and NIK, and its knockdown with silencing RNA impaired TNFα-induced NF-κB activation [4],[18]–[20].

Despite the (mostly in vitro) evidence for c-IAP regulation of NF-κB, primary cells from c-IAP1 and c-IAP2 knockout mice showed no obvious abnormalities in NF-κB activation [21],[22]. However, studies using synthetic “Smac mimetics” that induce the proteasomal degradation of c-IAP1 and c-IAP2, or siRNA to knock down the remaining c-IAP molecule expressed in cells from c-IAP1- and c-IAP2-deficient mice, have suggested that these two proteins may work redundantly to promote TNF-α-induced NF-κB activation and inhibit spontaneous non-canonical NF-κB activation [18],[20],[23]–[25]. Binding of c-IAPs to TRAF2 brings them into proximity with TRAF3-associated NIK. The result is a repressive complex that causes ubiquitination and degradation of NIK and maintains non-canonical NF-κB signaling in a basal state [3],[26],[27]. Consistent with this, tandem deletions of the c-IAPs have been associated with increased non-canonical NF-κB signaling and the development of multiple myeloma [28],[29], and conditional deletion of TRAF2 and TRAF3 results in stabilization of NIK, increased non-canonical signaling, and B cell hyperplasia [30]–[33].

MALT (mucosal associated lymphoid tissue) lymphomas are indolent neoplasms that have cytological features and bear cell surface markers of marginal zone B cells, and typically invade epithelial organs such as the gut and lung [34]–[36]. The molecular events that contribute to MALT lymphomagenesis are not well understood, but it is thought to involve the constitutive activation of NF-κB [37]. A variety of chromosomal abnormalities are associated with this disease; the most prevalent is a translocation, t(11;18)(q21;q21), that results in the production of a fusion protein containing the NH_2_-terminal (BIR-containing) fragment of c-IAP2 and the COOH-terminal portion of MALT1, a paracaspase involved in antigen receptor signaling [38]–[40]. Ectopic expression of this fusion protein in cell lines activates NF-κB [41], and transgenic overexpression in mice results in an increase in marginal zone B cells [42]. It is thought that the fusion protein activates NF-κB via the canonical signaling pathway [43]–[46]. The relevance of the different domains in the c-IAP2/MALT1 fusion protein to the development of MALT lymphoma has not been addressed.

Here we investigate the mechanism by which the c-IAP2/MALT1 fusion protein contributes to the development of MALT lymphoma. Ectopic expression of the fusion protein in cell lines activated both the canonical and non-canonical NF-κB signaling pathways, the former but not the latter being dependent on the MALT1 paracaspase activity. Expression of a mutant c-IAP2 that, like the c-IAP2 portion of the fusion protein, lacks E3 activity activated non-canonical but not canonical NF-κB. Knockin mice expressing this same c-IAP2 mutant in lieu of the wild type gene accumulated abnormal B-cells that had elevated non-canonical but not canonical NF-κB signaling, a cell-autonomous survival advantage in vivo, and other features of MALT lymphomas. The many points of similarity between mice expressing a c-IAP2 E3-inactive mutant and patients expressing a c-IAP2 E3-inactive MALT1 fusion protein suggests that the loss of this activity activates non-canonical NF-κB and predisposes to malignancy.

## Results

### Loss of c-IAP2 E3 Activity and Non-Canonical NF-κB Activation by the c-IAP2/MALT1 Fusion Protein

**Figure 1:**
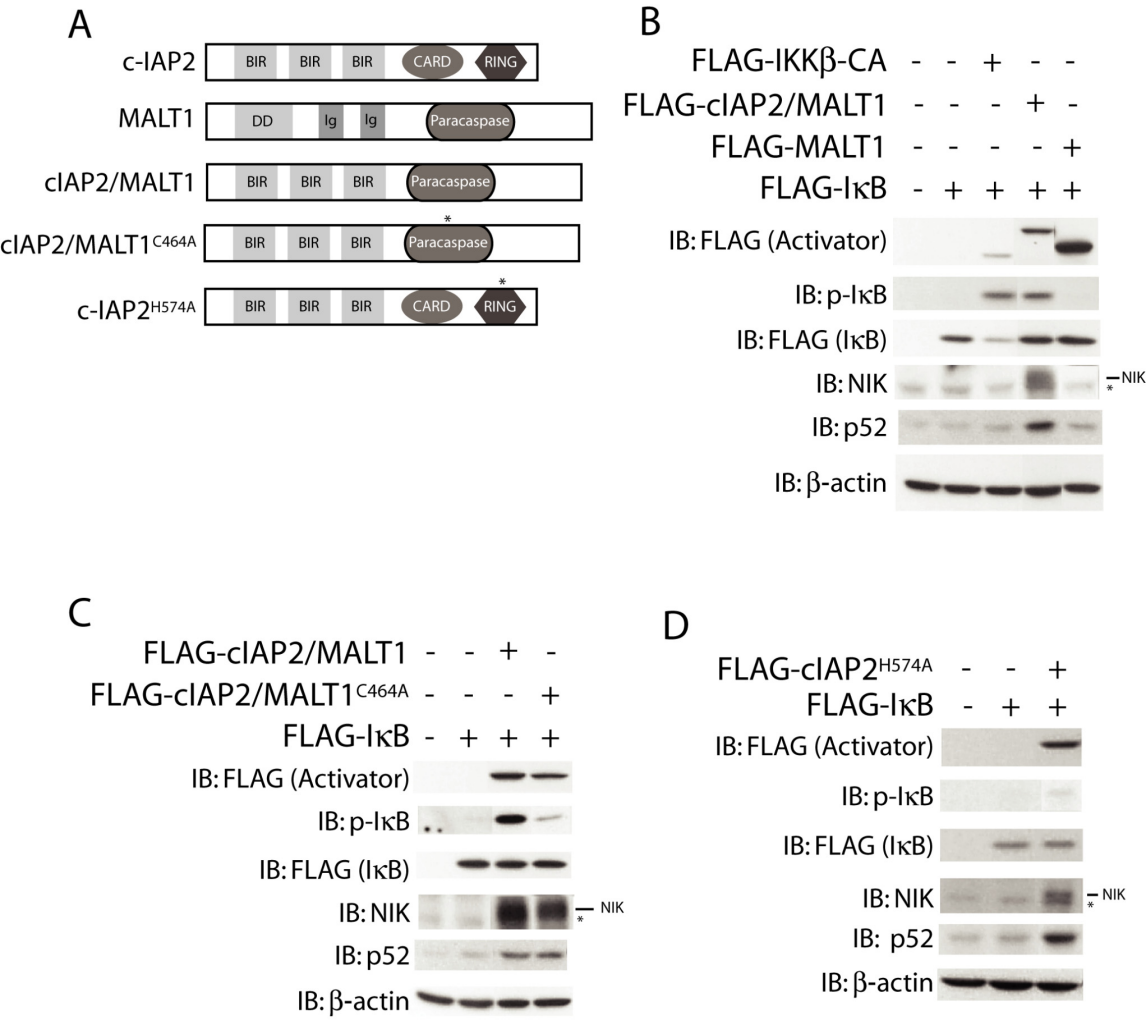
c-IAP2/MALT1 fusion protein activates canonical and non-canonical NF-κB signaling. (A) Schematic of c-IAP2, MALT1, c-IAP2/MALT fusion protein, and other mutants used. * denotes the location of the inactivating mutations. (B, C, and D) c-IAP1/MALT fusion protein activates canonical and non-canonical NF-κB signaling pathways. Lysates prepared from 293T cells that had been transfected with the indicated cDNAs for 24 h were immunoblotted with anti-FLAG, anti-phospho-IκB, anti-NIK, and anti-p52 antibodies. Because of differences in molecular weight, one can use anti-FLAG to detect and distinguish between the NF-κB activating proteins (Activator: IKKβ-CA, c-IAP2/MALT1, c-IAP2^H570A^, and MALT1) and IκB. Some lanes were reordered for clarity. β-actin expression was used as a loading control. * denotes a non-specific band.

Ectopic expression of the c-IAP2/MALT1 fusion protein causes p65 to translocate to the nucleus, evidence of canonical NF-κB activation [43]. We assessed the mechanism of NF-κB induction in 293T cells transfected with the fusion protein (Figure 1A and 1B). Expression of the c-IAP2/MALT1 fusion protein induced IκB phosphorylation, as did a constitutively active form of IKKβ (IKKβ-CA). Unlike IKKβ-CA, however, c-IAP2/MALT1 resulted in little if any IκB degradation, suggesting that it is a much less potent activator of canonical signaling. Notably, the c-IAP2/MALT1 fusion protein, but not IKKβ-CA, also increased the levels of NIK and p52, hallmarks of non-canonical signaling. Expression of MALT1 did not induce IκB phosphorylation or degradation, or increase NIK or p52. Therefore, the fusion protein can trigger both arms of the NF-κB signaling cascade.

The MALT1 portion of the c-IAP2/MALT1 fusion protein has paracaspase activity, and it has been shown that expression of an inactivating mutation resulted in approximately 2-fold less NF-κB reporter activity than the paracaspase-active form [47]. We compared NF-κB activation in 293T cells transfected with the native sequence or paracaspase-inactive (c-IAP2/MALT1^C464A^) c-IAP2/MALT1 cDNA (Figure 1C). The canonical pathway, as judged by IκB phosphorylation, was markedly reduced by the mutation, but increases in the non-canonical pathway components NIK and p52 were unaffected. The fusion protein lacks the c-IAP2 RING domain and therefore its E3 activity, and c-IAPs have been shown to ubiquitinate NIK and repress non-canonical NF-κB signaling [18],[26],[27]. In fact, expression of c-IAP2 lacking its c-terminal half, as occurs in c-IAP2/MALT1 fusion proteins, increased both NIK and p52 levels (Figure S1). To ask if this was due specifically to the loss of E3 activity, we expressed c-IAP2 in which a RING histidine that is critical for E3 activity was replaced by alanine (c-IAP2^H574A^), but the protein was otherwise intact (Figure 1A) [48],[49]. Expression of c-IAP2^H574A^ induced little if any IκB phosphorylation but increased NIK and p52 levels (Figures 1D and S1). These results show that the c-IAP2/MALT1 fusion protein activates both the canonical and non-canonical NF-κB signaling cascades and that there are two distinct mechanisms. The finding that expression of E3-defective c-IAP2 (Figure 1D) but not MALT1 (Figure 1B) activated non-canonical NF-κB raised the possibility that a similar mechanism might account for non-canonical NF-κB activation by the c-IAP2/MALT1 fusion protein.

### Generation of the c-IAP2^H570A^ Knockin Mice

**Figure 2:**
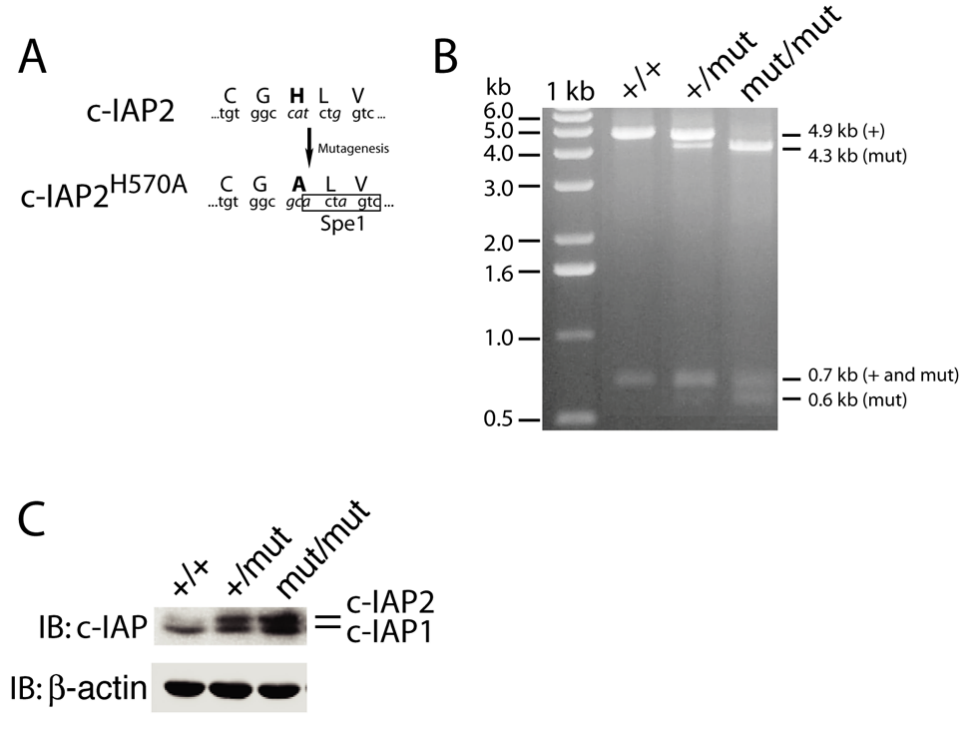
Generation of c-IAP2^H570A/H570A^ mice. (A) Site-directed mutagenesis of the Zn^2+^-coordinating histidine residue in the RING domain of c-IAP2. Lower and upper case letters denote the nucleotide and corresponding amino acid sequence, respectively, before and after mutagenesis. The bolded amino acids highlight the histidine to alanine replacement, and the italicized nucleotides highlight the nucleotide substitutions. The box depicts the novel Spe1 restriction endonuclease site. (B) Spe1 endonuclease digestion of PCR products amplified from wild type (+/+), c-IAP2^+/H570A^ (+/mut), and c-IAP2^H570A/H570A^ (mut/mut) tail DNA. SpeI digestion of the PCR fragment amplified from the wild type (+) allele generates 4.9 and 0.7 kb fragments and the c-IAP2^H570A^ (mut) allele 4.3, 0.7, and 0.6 kb fragments. (C) c-IAP expression in +/+, +/mut, and mut/mut splenocytes was determined by immunoblot using an antiserum that recognizes both c-IAP1 and c-IAP2. β-actin expression was used as a loading control.

To investigate the consequences of expressing c-IAP2 lacking E3 activity in vivo, we generated gene-targeted knockin mice that express an E3-inactive mutant of c-IAP2 (c-IAP2^H570A^) under the control of the native regulatory regions (Figure 2A). ES cells that had integrated the targeting vector were used to generate chimeric mice that were crossed to the C57BL/6 background. The presence of the H570A substitution in F1 offspring and subsequent generations was assessed by long-template PCR followed by Spe 1 restriction endonuclease digestion. The expected fragment sizes generated from the wild type allele are 4.9 and 0.7 kb, and those from the c-IAP2^H570A^ allele are 4.3 and 0.6 kb (Figure S2 and Figure 2B). Acquisition of the mutant allele in c-IAP2^+/H570A^ and c-IAP2^H570A/H570A^ mice caused the appearance of shorter fragments in a gene dose-dependent manner.

Mutation of the Zn^2+^-coordinating histidine in the c-IAP1, c-IAP2, and XIAP RING domains [48],[49] prevents autoubiquitination and results in increased protein levels in cells transiently expressing the corresponding cDNAs. Furthermore, under physiologic conditions c-IAP1 downregulates c-IAP2 protein levels by trans-ubiquitination and proteasomal degradation [21]; there does not seem to be a reciprocal regulation of c-IAP1 by c-IAP2 [22]. To determine how c-IAP2 E3 activity might affect c-IAP levels, splenocyte lysates were immunoblotted with an antiserum that recognizes both c-IAP2 and c-IAP1 (Figure 2C) [50]. The antibody detected a doublet in wild type cells, the upper and fainter band being c-IAP2 and the lower and more prominent being c-IAP1 [21]. There was a marked increase in c-IAP2 expression in c-IAP2^+/H570A^ cells and an even greater increase in c-IAP2^H570A/H570A^ cells. In contrast, there was only a small increase in the level of c-IAP1. We compared the susceptibility of wild type c-IAP2 and the RING-less c-IAP2/MALT1 fusion protein to ubiquitination-dependent degradation. Consistent with a previous report [51], only levels of c-IAP2 increased in response to proteasome inhibition, indicating that the lack of E3 activity also stabilizes the fusion protein (Figure S3). Given that c-IAP2 expression is also regulated by c-IAP1-mediated ubiquitination [21], these results indicate that the combined activity of the c-IAPs is required to maintain c-IAP2 at physiologic levels.

### B Cell Hyperplasia, Marginal Zone B Cell Accumulation, and Enlarged GALT in c-IAP2^H570A/H570A^ Mice

**Figure 3:**
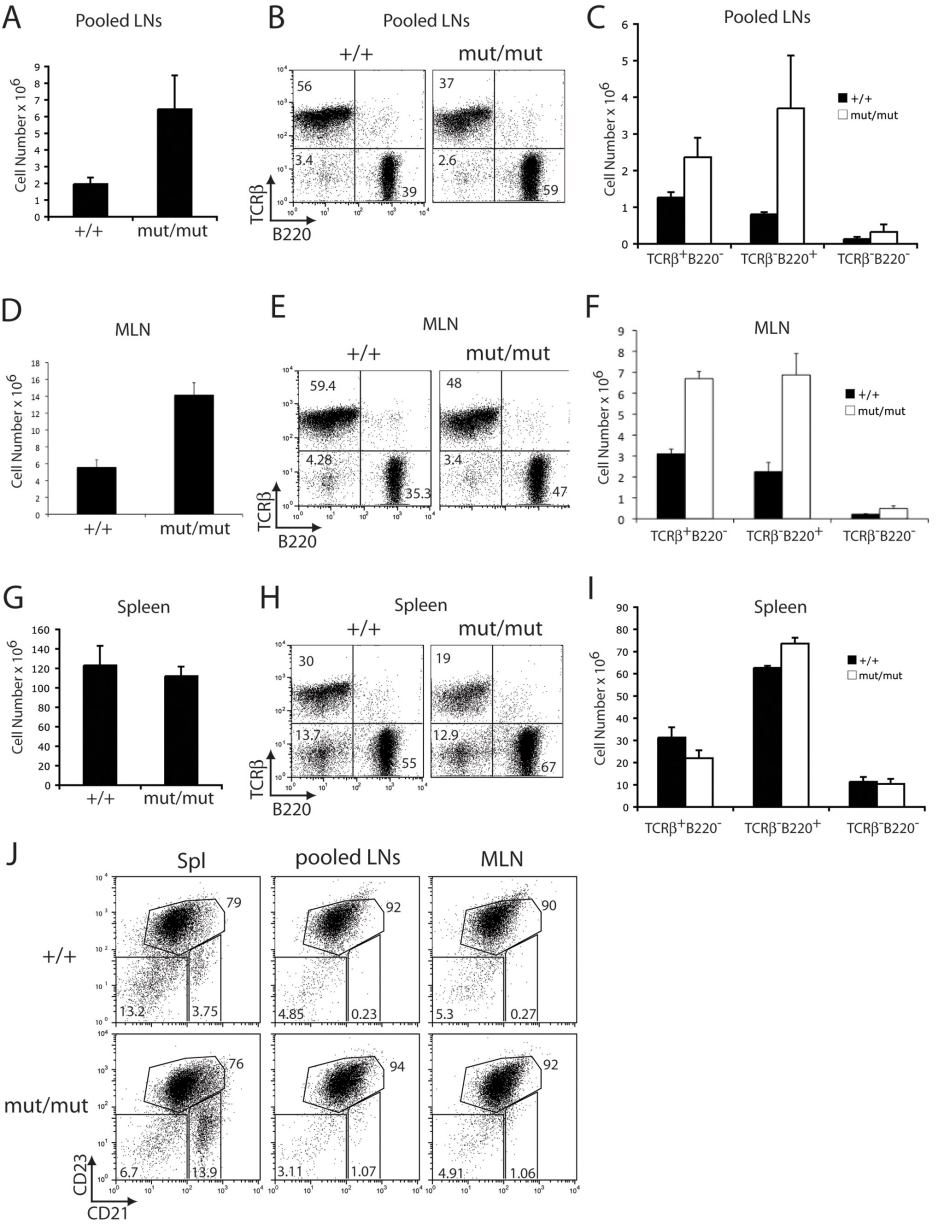
Secondary lymphoid homeostasis in 6–7-mo-old c-IAP2^H570A/H570A^ mice. Cellularity of pooled axial, brachial, superficial cervical, and inguinal lymph nodes (Pooled LNs) (A), mesenteric lymph node (MLN) (D), and spleen (G) in wild type (+/+; *n* = 3) and c-IAP2^H570A/H570A^ (mut/mut; *n* = 3) mice. The error bars represent the standard error of the mean. T (TCRβ^+^B220^−^), B (TCRβ^−^B220^+^), and non-T and non-B cell (TCRβ^−^B220^−^) distribution in pooled LNs (B), MLN (E), and spleen (H) of +/+ and mut/mut mice determined by flow cytometry. Numbers represent the percentage of cells in each quadrant. Absolute number of T, B, and non-T/non-B cells in pooled LNs (C), MLN (F), and spleen (I) of +/+ and mut/mut mice. For the pooled lymph node, the absolute number was divided by the total number of lymph nodes harvested. The error bars represent the standard error of the mean. (J) Distribution of immature (CD23^−^CD21^−^), follicular (CD23^+^CD21^int^), and marginal zone (CD23^−^CD21^+^) B cells (gated on B220^+^ cells) in spleen (Spl), pooled LNs, and MLN. Numbers represent the percent positive in each gate.

**Figure 4:**
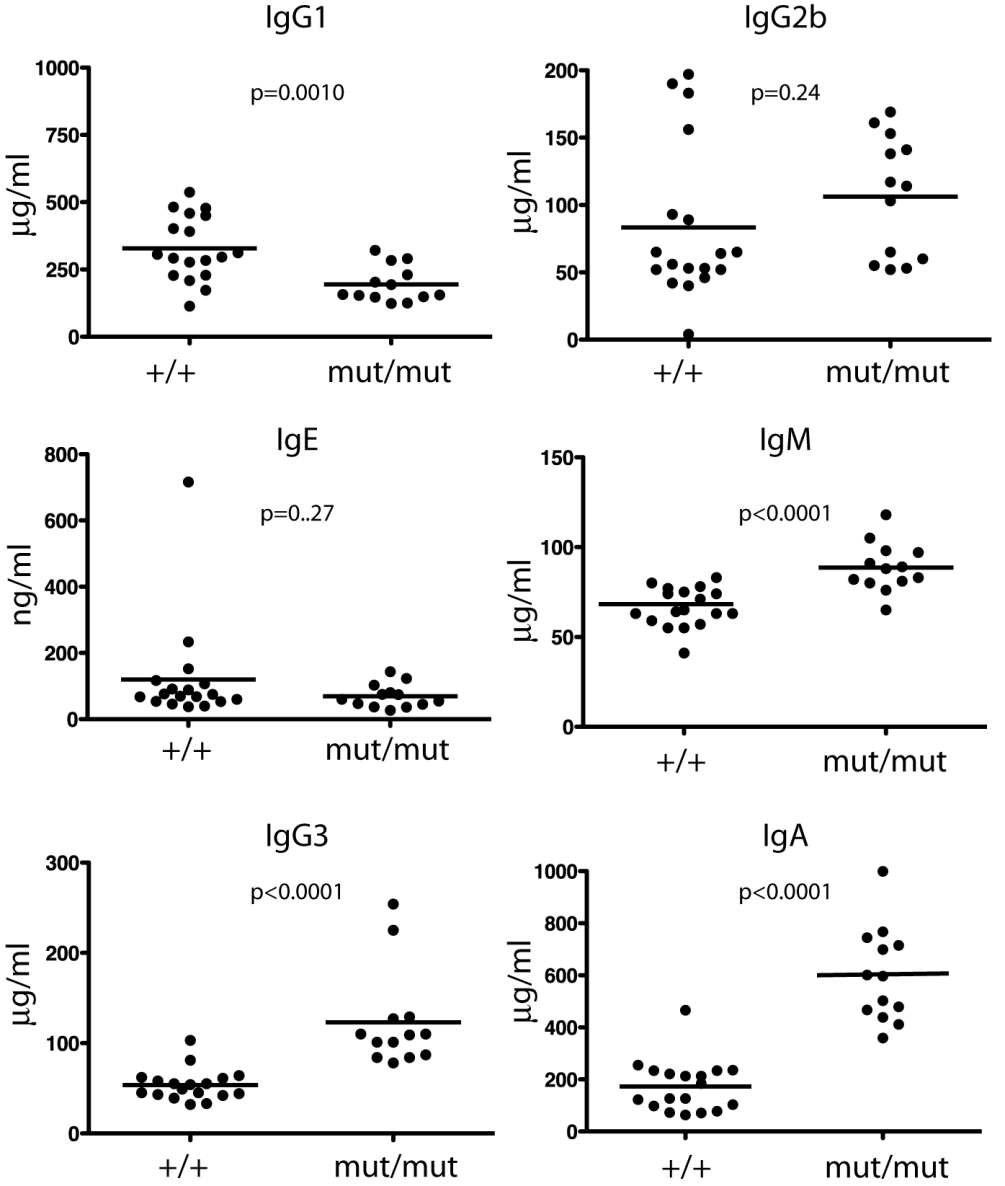
Serum immunoglobulin levels in c-IAP2^H570/H570A^ mice. Wild type (+/+) and c-IAP2^H570A/H570A^ (mut/mut) mice were 8–10-wk-old, each dot represents the titer for an individual mouse, and the horizontal lines indicate the mean titer for each genotype.

Homozygous c-IAP2 knockin mice were viable, fertile, and displayed no obvious phenotypic abnormalities. Analysis of peripheral lymphoid organs in 6–7-month-old c-IAP2^H570A/H570A^ mice, however, revealed a number of abnormalities. Unlike the spleen, cell numbers of pooled peripheral lymph nodes (axial, brachial, superficial cervical, and inguinal) as well as mesenteric lymph nodes were markedly increased (Figure 3G, A, and D). There was a reduction in the percentage of T cells with a corresponding increase in the percentage of B (B220^+^) cells (Figure 3B, E, and H). The result was approximately a 5-fold and 4-fold increase in the absolute number of pooled and mesenteric lymph node B cells, respectively, and a smaller (2-fold) increase in T cell number (Figure 3C and F). The CD4^+^∶CD8^+^ T cell ratio in c-IAP2^H570A/H570A^ mice was normal (unpublished data). c-IAP2^H570A/H570A^ lymphocytes had an unactivated phenotype, with normal levels of B7.1 and I-A^b^ (B cells) and CD25 and CD69 (T cells) (unpublished data). Two- to three-month-old c-IAP2^H570A/H570A^ mice also had increases in lymph node B cells, although to a lesser extent than older animals (Figure S4). Analysis of B and T cell precursors in bone marrow and thymus, respectively, revealed no abnormalities. Among splenic B cells there was reproducibly an approximately 3-fold increase in the percentage of cells with a marginal zone phenotype (CD21^hi^CD23^−^), with a compensatory decrease in the percentage of follicular (CD21^int^CD23^hi^) and immature (CD21^−^CD23^−^) B cells (Figure 3J). Although lymph nodes normally have few B cells with a marginal zone phenotype [52], there was a small increase in these cells in c-IAP2^H570A/H570A^ lymph nodes. Circulating IgA was increased approximately 3-fold in c-IAP2^H570A/H570A^ mice, and there were highly statistically significant increases in IgM and IgG3, and a reduction in IgG1 as well (Figure 4). No statistically significant changes were found in IgG2b and IgE levels.

**Figure 5:**
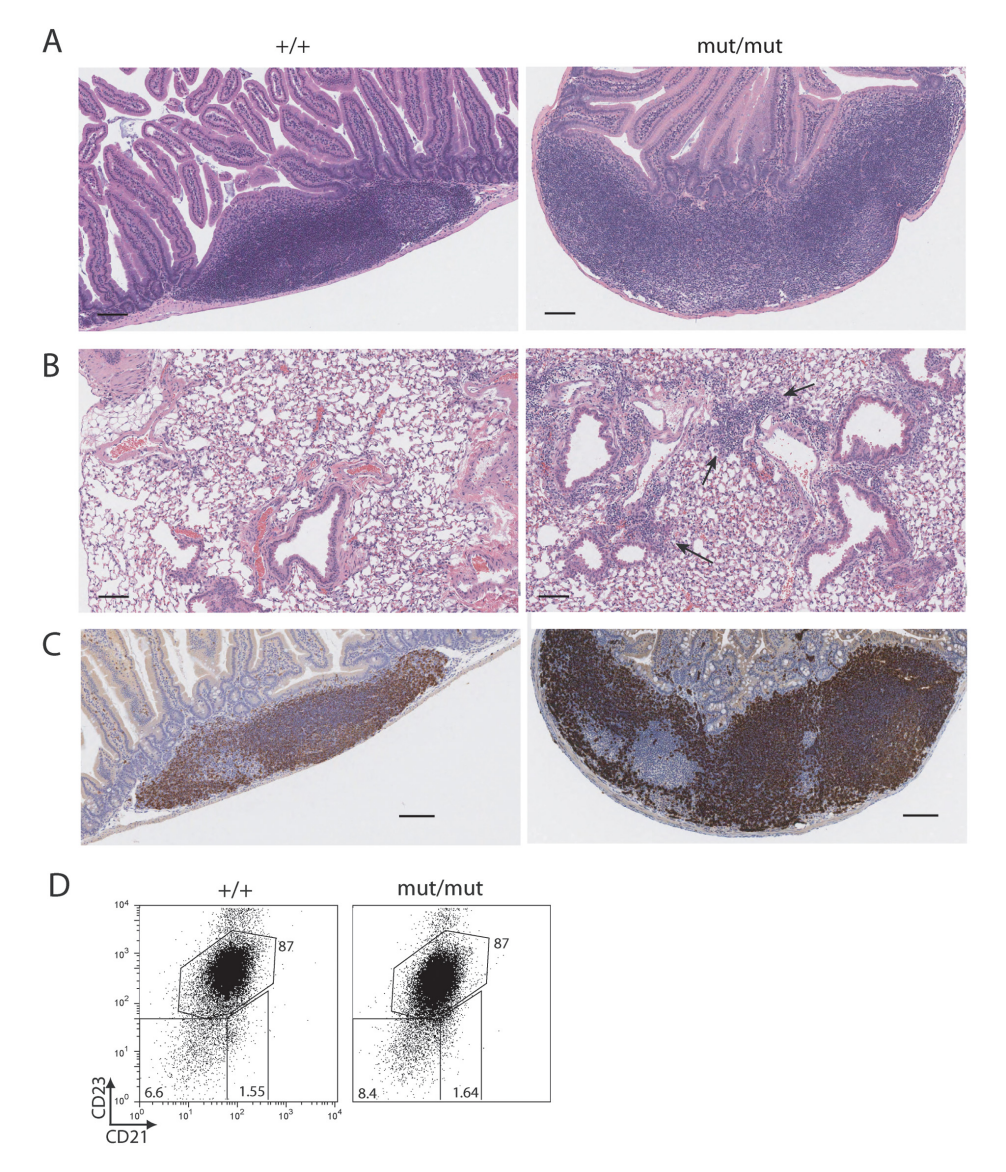
Lymphocytic infiltrates in the lungs and GALT hyperplasia in c-IAP2^H570A/H570A^ mice. Sections of GALT (A) and lung (B) from wild type (+/+) and c-IAP2 knockin (mut/mut) mice stained with hematoxylin and eosin. Both tissues are presented at 8× magnification and the bar denotes 100 µm. Arrows highlight the areas containing the infiltrating lymphocytes in the lungs of c-IAP2 ^H570A/H570A^ mice. (C) B cell distribution in GALT of +/+ and mut/mut mice. (D) Distribution of immature (CD23^−^CD21^−^), follicular (CD23^+^CD21^int^), and marginal zone (CD23^−^CD21^+^) B cells (gated on B220^+^ cells) in GALT of +/+ and mut/mut mice. Numbers represent the percent positive in each gate.

B cell hyperplasia, particularly of marginal zone B cells, in gut-associated lymphoid tissue (GALT) and lung is a feature of MALT lymphomas [34],[36]. Gross examination revealed that c-IAP2^H570A/H570A^ mice had enlarged GALT and mesenteric lymph nodes, which was confirmed by histological evaluation (Figure 5A). There were also mild to moderate lymphocytic infiltrates in the lung (Figure 5B), with no evidence of neoplasia in either organ. Despite the increased size of the GALT in c-IAP2^H570A/H570A^ mice, immunohistochemistry and flow cytometric analysis of both wild type and c-IAP2^H570A/H570A^ GALT revealed primarily B cells with a follicular phenotype (Figure 5C and 5D), organized T-cell-enriched areas (compare Figure 5C with Figure S5), and no evidence of cellular activation (unpublished data). The lymphocytic infiltrates in the lungs of the c-IAP2 knockin mice also consisted of B cells and T cells (unpublished data). Taken together, these results demonstrate that mice with catalytically inactive c-IAP2 acquire a lymphoid phenotype that shares many features with MALT lymphomas.

### Enhanced Survival and Proliferation of c-IAP2^H570A/H570A^ B Cells In Vitro and In Vivo

**Figure 6:**
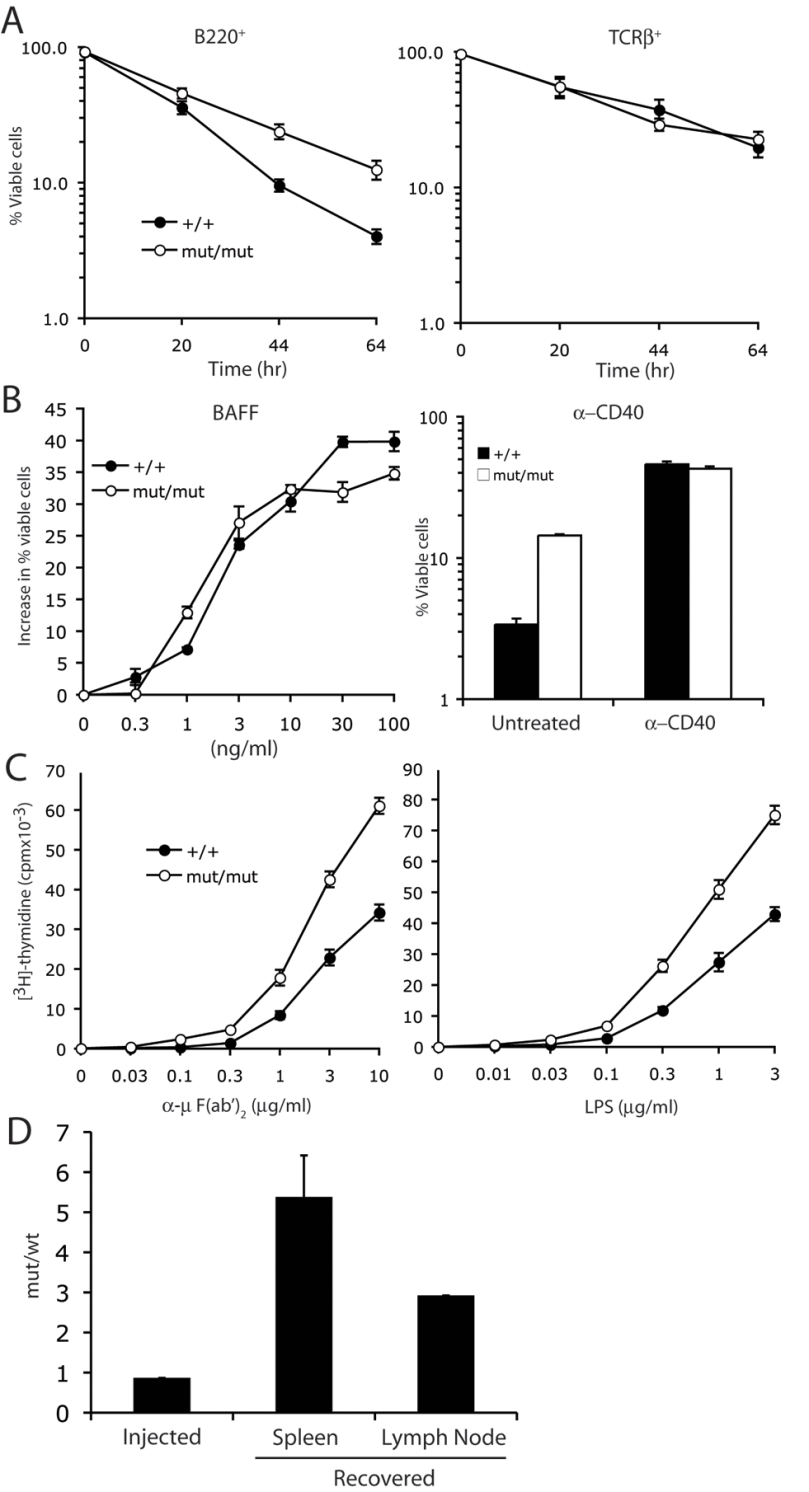
c-IAP2^H570A/H570A^ B cells are more sensitive to proliferative stimuli and survive longer in vitro. (A) Splenocytes from wild type and c-IAP2^H570A/H570A^ mice were cultured for the indicated times and the percentage of viable B and T cells was quantitated by staining with anti-B220, anti-TCRβ, and 7AAD. The experiment was performed in triplicate and the error bars represent the standard error of the mean. (B) Purified B cells were incubated with or without BAFF or agonistic anti-CD40 for 66 h and cell viability was determined as in (B). For the assessment of BAFF sensitivity, the percent viability of untreated cells was subtracted from the percent viability at each concentration of BAFF and the results are displayed as the increase in percent viable cells. (C) IgM- and LPS-induced proliferative responses of wild type (+/+) and c-IAP2^H570A/H570A^ (mut/mut) B cells. Purified B cells were cultured in vitro with the indicated concentrations of anti-μ (Fab′)_2_ or lipopolysaccharide (LPS) for 48 h, pulsed with ^3^H-thymidine, and harvested 18 h later. The experiment was performed in triplicate and the error bars represent the standard error of the mean. (D) Adoptive transfer of equal numbers of splenic wild type (wt; CD45.1^+^) and c-IAP2^H570A/H570A^ (mut; CD45.2^+^) B cells into RAG2-deficient mice. Forty-five days later splenocytes and lymphocytes were prepared and the ratio of mut/wt B cells recovered was calculated by dividing the percentage of mut (B220^+^CD45.2^+^) cells by the percentage of wt (B220^+^CD45.1^+^) cells.

The increase in B cell numbers in vivo could be due to decreased death, increased expansion, or a combination. Susceptibility to cell death was determined by culturing splenocytes in the absence of growth or survival factors and quantifying cell viability of B220^+^ and TCRβ^+^ cells by measuring 7-AAD incorporation (Figure 6A). c-IAP2 knockin B cells died more slowly than wild type cells, with 10%–15% still viable even after 64 h, compared to 3% for wild type cells. Addition of BAFF or agonistic anti-CD40 partially rescued the survival of B cells of both genotypes with similar dose-response curves (Figure 6B and unpublished data). There was no difference between the genotypes with regard to T cell survival (Figure 6A). Proliferative ability was addressed by stimulating purified B cells with anti-μ F(ab′)_2_ or lipopolysaccharide (LPS) and measuring ^3^H-thymidine incorporation (Figure 6C). c-IAP2^H570A/H570A^ B cells had enhanced responses to both stimuli, with approximately a 3-fold shift in the dose response curve toward lesser concentrations of stimulus compared to wild type cells. During the course of the proliferation assays there were no differences between the two genotypes with regard to cell death (unpublished data). To determine if these in vitro observations correspond to B cell behavior in vivo, experiments were performed in which a mixture of wild type and c-IAP2 knockin splenic B cells was adoptively transferred into RAG2-deficient mice (Figure 6D). Although equal numbers of cells of each genotype were injected, after 45 d a 3-fold (lymph node) to 5-fold (spleen) preponderance of c-IAP2 knockin B cells was observed. These results show that the absence of c-IAP2 E3 activity in B cells results in a cell-intrinsic abnormality that increases their capacity to survive and/or proliferate in vitro and in vivo.

### Spontaneous Non-Canonical NF-κB Activation in c-IAP2^H570A/H570A^ Cells

**Figure 7:**
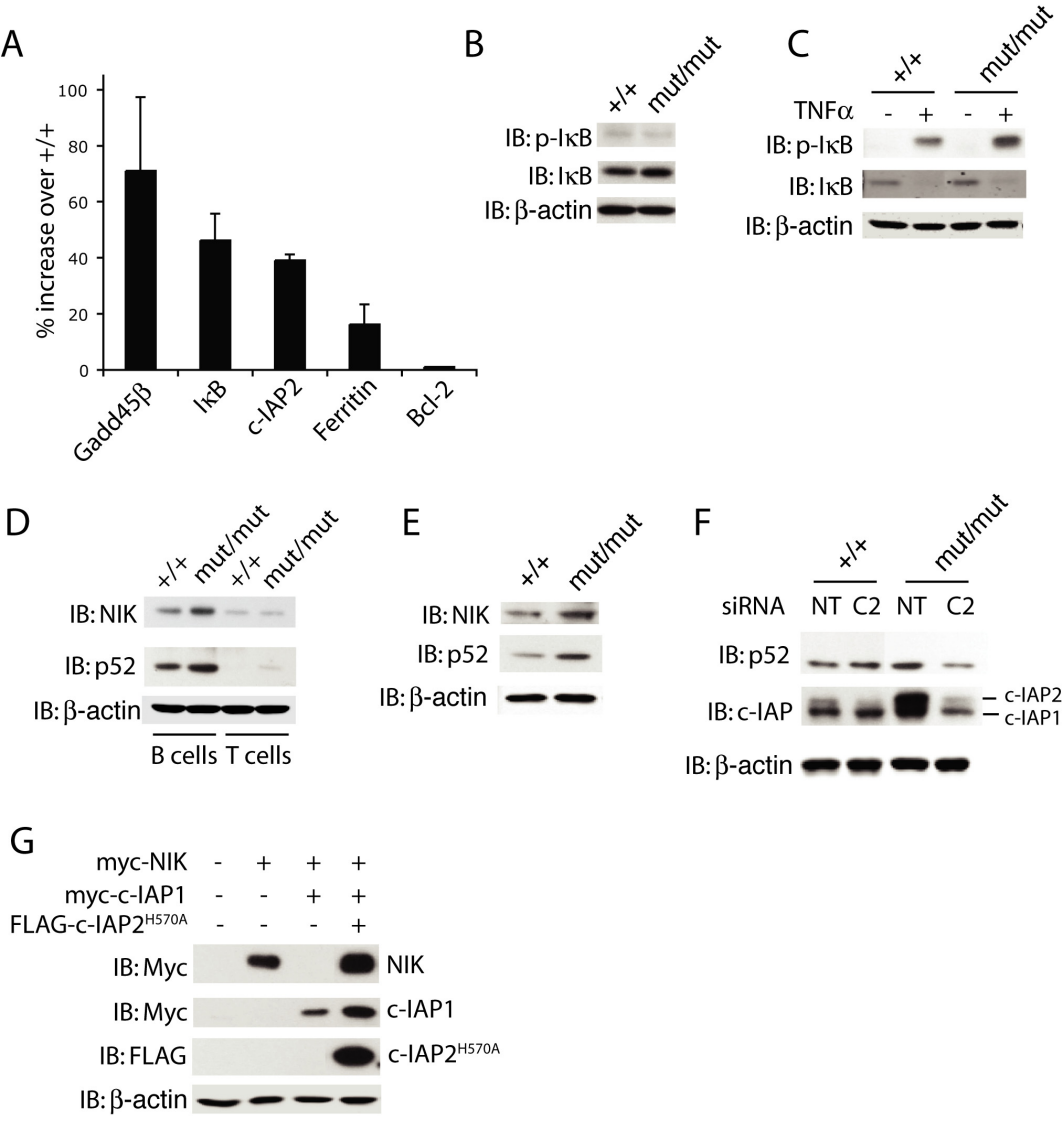
Elevated non-canonical NF-κB activation in c-IAP2^H570A/H570A^ cells. (A) NF-κB target gene expression in WT (+/+) and c-IAP2^H570A/H570A^B cells determined by real-time PCR. Bars represent the mean increase of each mRNA in c-IAP2^H570A/H570A^ cells from three independent experiments. The error bars represent the standard error of the mean. (B) Immunoblotting of phospho-IκB and IκB in lysates of +/+ and c-IAP2^H570A/H570A^ (mut/mut) B cells. β-actin expression was used as a loading of control. (C) Immunoblotting of phospho-IκB and IκB levels in lysates of unstimulated +/+ and mut/mut MEFs as in (B). Lysates of cells stimulated with TNF-α (10 ng/ml for 5 min) were blotted in parallel as a positive control for the phospho-IκB antibody. (D) Immunoblotting of NIK and p52 in lysates prepared from +/+ and mut/mut B and T cells. β-actin expression was used as a loading of control. (E) Immunoblotting of NIK and p52 in lysates prepared from +/+ and mut/mut MEFs as in (D). (F) Knockdown of c-IAP2 in +/+ and mut/mut MEFs. Cells were transfected with non-targeting (NT) or c-IAP2 siRNA (C2) for 24 h, harvested, and lysed. c-IAP2 and p52 levels were determined by immunoblot. β-actin expression was used as a loading of control. The lanes have been reordered for clarity. (G) 293T cells were transfected with expression vectors containing the indicated cDNAs and immunoblotted for the respective peptide tags and β-actin.

Ectopic expression of a c-IAP2/MALT1 fusion protein spontaneously activates NF-κB, as does depletion of c-IAPs with Smac mimetics or silencing siRNAs [18],[25],[26],[38],[41],[53],[54]. We therefore asked if selective loss of c-IAP2 E3 activity, in an otherwise physiological setting, affects NF-κB. Quantitative RT-PCR found that transcripts for NF-κB-responsive genes encoding GADD45β, IκB, c-IAP2, and ferritin heavy chain were elevated in c-IAP2^H570A/H570A^ B cells (Figure 7A) [15],[55]–[57]. There was no increase, however, in the expression of Bcl-2, a gene product that has been reported to increase in response to canonical but not non-canonical NF-κB activation [58],[59], raising the possibility that NF-κB activation in c-IAP2^H570A/H570A^ B cells was pathway-specific. Activation of the canonical pathway was assessed by measuring IκB levels and its state of phosphorylation. IκB levels were similar to or perhaps slightly increased in c-IAP2^H570A/H570A^ B cells (Figure 7B) and murine embryonic fibroblasts (MEFs) (Figure 7C) compared to wild type cells. More importantly, there was no increase in spontaneously phosphorylated IκB in c-IAP2^H570A/H570^ cells, arguing against spontaneous canonical NF-κB activation. In contrast, the levels of both NIK and p52 were elevated in knockin B cells (Figure 7D) and MEFs (Figure 7E). The levels of TRAF2 and TRAF3, two components of a c-IAP-containing inhibitory complex thought to degrade NIK [26],[27], were unaffected by the loss of c-IAP2 E3 activity (unpublished data). In T cells, the amount of NIK was lower in wild type T than wild type B cells, and there was little increase in T cells expressing E3-inactive c-IAP2 (Figure 7D). There was correspondingly little increase in p52, although a small amount was detected in c-IAP2^H570A/H570A^ T cells. Together, these results indicate the E3 activity of c-IAP2 is required to inhibit constitutive non-canonical NF-κB activation in B cells, MEFs, and to a much lesser degree, T cells.

Although the E3 activity of c-IAP2 is absent in both c-IAP2^−/−^ and c-IAP2^H570A/H570A^ cells, only the latter has increased spontaneous NF-κB activation. Because c-IAP1 also binds TRAF2, which is essential for c-IAP-mediated repression of the non-canonical signaling cascade [26], the results are consistent with the possibility that the E3-defective c-IAP2 competes with endogenous c-IAP1. In fact, c-IAP2^H570A/H570A^ is able to bind TRAF2 at least as well as the wild type protein (Figure S6). To ask if the c-IAP2 RING mutant interfered with endogenous c-IAP1, c-IAP2-specific siRNA was used to knock down c-IAP2 in wild type and c-IAP2 knockin MEFs (Figure 7F). As seen in splenocytes (Figure 2C), there was a large increase in c-IAP2 and a small increase in c-IAP1 levels in c-IAP2 knockin MEFs (Lanes 1 and 3). Transfection of wild type MEFs with c-IAP2 siRNA specifically reduced c-IAP2 but had little if any effect on the levels of p52. In contrast, knockdown of c-IAP2 in c-IAP2^H570A/H570A^ MEFs resulted in a substantial reduction of p52 levels. To determine if c-IAP2^H570A^ interferes with c-IAP1-mediated ubiquitination/degradation of NIK, 293T cells were co-transfected with NIK and c-IAP1, with or without c-IAP2^H570A^ (Figure 7G). Consistent with previous reports [18],[26], expression of c-IAP1 reduced NIK to undetectable levels; this was prevented by co-expression of E3-inactive c-IAP2. Thus, the E3-defective c-IAP2^H570A^ can inhibit constitutive c-IAP1-mediated ubiquitination/degradation of NIK and de-repress the non-canonical signaling cascade.

## Discussion

Unmanipulated mice deficient for c-IAP1 and c-IAP2 have no obvious phenotypic abnormalities [21],[22], which has made it difficult to ascribe a physiologic role to these proteins in vivo. Recent studies have suggested that redundancy among the c-IAPs, at least with regard to NF-κB activation, could account for the lack of apparent abnormalities [18],[25],[26]. If so, this could be an even bigger factor in c-IAP1 knockout mice, in which c-IAP2 levels are elevated because it is no longer ubiquitinated by c-IAP1 and targeted for degradation [21]. c-IAP1 is not elevated in cells from c-IAP2-deficient mice [22], suggesting that even normal c-IAP1 levels are sufficient to compensate for the loss of c-IAP2. In contrast to the c-IAP2 knockout animals, we have found that substitution of wild type c-IAP2 with an E3-defective point mutation does result in constitutive NF-κB activation and abnormal B cell accumulation. This is likely because the endogenous c-IAP1 is unable to compensate for the lack of c-IAP2 E3 activity. The N-terminal BIR-containing region of both proteins binds to TRAF2, a prerequisite for c-IAP-mediated NIK ubiquitination [18],[26]. Furthermore, only one c-IAP molecule can bind one TRAF2 trimer at a time [60]. We found that overexpressed c-IAP2^H570A/H570A^ interferes with c-IAP1-mediated degradation of NIK and that knockdown of the c-IAP2 mutant restored repression of non-canonical NF-κB. These data argue that the mutant c-IAP2 prevented c-IAP1 from associating with the repressive complex. The c-IAP2^H570A/H570A^ mice therefore represent an example in which replacement of the endogenous gene with an inactive form, but not a complete knockout, can reveal normal function.

Abnormal B cell expansion has been observed in a number of animal models in which NF-κB activity is chronically elevated. For example, overexpression of B cell activating factor (BAFF) or NIK, both of which lead to non-canonical NF-κB activation, results in B cell hyperplasia with increased numbers of CD23^lo^CD21^hi^ B cells [59],[61]. Similarly, mice lacking either TRAF2 or TRAF3 in B cells have elevated non-canonical NF-κB, an expanded B cell compartment, increased numbers of cells with a marginal zone phenotype, and elevated serum immunoglobulins [31]–[33]. TRAF2 and TRAF3 are adaptor molecules downstream of BAFF receptors that constitutively form a complex with c-IAP1, c-IAP2, and NIK [26],[27]. These associations result in c-IAP-dependent ubiquitination of NIK and its proteasomal degradation, which is thought to maintain the non-canonical NF-κB activation pathway in a basal state. Although we found no alterations in expression of TRAF2 and TRAF3 in c-IAP2^H570A/H570A^ mice, NIK levels and NF-κB activity were increased, and the mice developed age-dependent B cell hyperplasia in a manner similar to BAFF and NIK transgenic mice, or TRAF2 and TRAF3 knockout mice [31]–[33],[59],[61]. The data are all consistent with the notion that basal ubiquitination of NIK by c-IAP2 is an important mechanism for regulating constitutive NF-κB activity and B cell homeostasis.

It is widely believed that the c-IAP2/MALT1 protein is pathogenic because it activates the canonical NF-κB signaling pathway [37]. A variety of mechanisms have been suggested, including proteolytic cleavage of A20, a negative regulator of NF-κB activation, ubiquitination of NEMO, binding of the fusion protein to lysine 63-linked polyubiquitinated NEMO, and the failure of the fusion protein to degrade Bcl-10 [37]. However, a potential role for non-canonical NF-κB activation has not been explored. We have found that the c-IAP2/MALT1 fusion protein activates both canonical and non-canonical signaling pathways, and activation of the latter in mice is sufficient to promote the development of features common to MALT lymphoma. Our results are in agreement with a report that overexpression of the fusion protein in 3T3 cells resulted in an NF-κB complex that was supershifted with antibodies to RelB [62]. Interestingly, introduction of a Bcl-10 transgene, which mimics the MALT lymphoma-associated t(1;12)(p22;q32) chromosomal translocation that deregulates Bcl-10, results in marginal zone B cell hyperplasia and elevated non-canonical as well as canonical NF-κB signaling [63]. It is noteworthy that mice lacking the COOH-terminal ankyrin domain of p100, which results in constitutive activation of p52, develop B cell hyperplasia and enlarged GALT. Thus, activation of the non-canonical pathway may be a major contributor to the development of MALT lymphoma. The development of MALT lymphoma-like abnormalities in the c-IAP2 E3-defective mice raises a cautionary note that drugs that reduce c-IAP levels, such as SMAC mimetics, may have unintended side effects due to activation of non-canonical NF-κB signaling, especially if administered chronically.

## Materials and Methods

### Mice and Reagents

RAG2-deficient and CD45.1 congenic mice were obtained from the Jackson Laboratory. All restriction endonucleases were obtained from New England Biolabs. pCMV9 containing carboxy-terminal myc-tagged human NIK cDNA was obtained from Nobuhiko Kayagaki and Vishva Dixit (Genentech) and pRK5 containing Flag-tagged human c-IAP2 and c-IAP2/MALT1 was obtained from Xiaolu Yang (University of Pennsylvania). pRK5-Flag-tagged human c-IAP2^H574A^ and pRK5-Flag-tagged human c-IAP2/MALT1^C464A^ were generated by site directed mutagenesis using the primers 5′-GTCCATAGTGTTTATTCCTTGTGGTCATCTAGTAGTATGCAAAGATTGTGC-3′, 5′-GCACAATCTTTGCATACTACTAGATGACCACAAGGAATAAACACTATGGAC-3′, 5′-GACTTAATGTGTTCTTATTGGATATGGCTAGGAAAAGAAATGACTACGATGATAC-3′, 5′-GTATCATCGTAGTCATTTCTTTTCCTAGCCATATCCAATAAGAACACATTAAGTC-3′, respectively, and the QuickChange mutagenesis system from Stratagene. pRK5-Flag-tagged human c-IAP2^ΔCARD-RING^ was generated by cloning a PCR product amplified from human c-IAP2 cDNA into pRK5 that already contained cDNA encoding the Flag-tag using the primers 5′-GCTCGTGAATGCGGGATCCTCTAGAAACATAGTAGAAAACAGC-3′ and 5-GCTGCAACGTAAGCTTTCATTCATTTGATTCTTTTTCCTCAGTTGC-3′, BamH1 and HindIII. Presence of the mutations was confirmed by direct sequencing. pCMV-Tag2 murine c-IAP2 has been described [21]. GST-tagged murine c-IAP2 was obtained by subcloning into pGEX-6P-1 (Amersham). GST-tagged murine c-IAP2^H570A^ was generated by site directed mutagenesis using primers that have been described [21]. Myc-tagged murine c-IAP1 was obtained by subcloning into pCMV-Tag5 (Clontech). IKKβ-CA has been described [64]. pCMV4 containing Flag-tagged IκB cDNA was obtained from Dean Ballard (Vanderbilt University). The anti-c-IAP antibody was obtained from Herman Chung and Bob Korneluk (Apoptosis Research Center, Children's Hospital of Eastern Ontario), anti-NIK and anti-p52 from Cell Signaling Technologies, anti-phospho-IκB and anti-IκB from Santa Cruz, and anti-FLAG and anti-β-actin from Sigma. Anti-CD40 (HM40-3) was obtained from BD Biosciences. BAFF was obtained from Peprotech. The fluorescently labeled antibodies used for analysis of lymphoid populations in the thymus, bone marrow, lymph node, and spleen by flow cytometry were obtained from BD Biosciences. The Mouse Immunoglobulin Isotype Panel (Southern Biotech) was used to quantify the serum immunoglobulin titers for IgM, IgG1, IgG2b, IgG3, and IgA. The OptEIA Set Mouse IgE (BD Biosciences) was used to quantify the amount of serum IgE. The anti-B220/CD45R and anti-CD3 used from B and T cell immunohistochemistry were purchased from BD Biosciences and Serotec, respectively. The B cell and T cell enrichment kits were obtained from Stemcell Technologies. For some experiments B cells were purified using the Mouse B cell Recovery Column Kit from Cedarlane Laboratories Ltd. The primer sequences used in the quantitative PCR are as follows, GADD45β 5′ (5′-CTGCCTCCTGGTCACGAA-3′), GADD45β 3′ (5′-TTGCCTCTGCTCTCTTCACA-3′), IκB 5′ (5′-TCACGGAGGACGGAGACTCG-3′), IκB 3′ (TGGAGATGCTGGGGTGTGC), ferritin heavy chain 5′ (5′-GGAGTTGTATGCCTCCTACGTCT-3′), ferritin heavy chain 3′ (5′-TGGAGAAAGTATTTGGCAAAGTT-3′), c-IAP2 5′ (5′-TATTTGTGCAACAGGACATTAGGAGT-3′), c-IAP2 3′ (TCTTTCCTCCTGGAGTTTCCG), Bcl-2 5′ (5′-GTACCTGAACCGGCATCTG-3′), and Bcl-2 3′ (5′-GGGGCCATATAGTTCCACAA-3′). The HPRT primers have been described [65]. c-IAP2 siRNA has been described [26] and was modified to Stealth RNAi siRNA. The sequences of the oligonucleotides are 5′-AAGUGGUAGGGACUUGUGCUCAAAG-3′ and 5′-CUUUGAGCACAAGUCCCUACCACUU-3′.

### Gene Targeting and Generation of the c-IAP2^H570A/H570A^ Mice

The BamH1-EcoR1 and EcoR1-EcoR1 recombination arms used to generate the c-IAP2^H570A^ targeting construct were obtained from BAC-DNA (clone 239-13P; Research Genetics) using the respective endonucleases and subcloned into shuttle vectors. To insert the silent mutation in the neighboring leucine codon introducing a novel Spe1 restriction endonuclease site and then replace the histidine codon with an alanine codon, the BamH1-Ecor1 arm was sequentially mutagenized using mutagenic primers 5′-CATCGTGTTCATTCCCTGTGGCGCACTAGTCGTGTGCAAAGACTGCG-3′ and 5′-CGCAGTCTTTGCACACGACTAGTGCGCCACAGGGAATGAACACGATG-3′, and then 5′-CATTCCCTGTGGCCATCTAGTCGTGTGCAAAGACTGC-3′ and 5′-GCAGTCTTTGCACACGACTAGATGGCCACAGGGAATG-3′ using the QuickChange mutagenesis system from Stratagene. The presence of H570A in exon 9 and absence of other spontaneous mutations in the other exons were confirmed by direct sequencing. After subcloning both recombination arms into a vector containing a neomycin cassette flanked by two loxP recombination sites, the resultant targeting vector was linearized with Not1 and transfected into ES cells. Stable transfectants were screened by southern blotting and long-range polymerase chain reaction (LR-PCR) coupled with Spe1 restriction endonuclease digestion. The primers used to screen the c-IAP2^H570A/H570A^ mice were obtained from Invitrogen and their sequence was 5′ CGAAAAAGATGCCCATCTACTCAG-3′ and 5′-TATCCCTAAAATGTCATCCAATAAATAACAG-3′. The clone that had correctly integrated the targeting construct at the c-IAP2 locus was injected into blastocytes to generate chimeric mice. F_1_ offspring of the chimeric mice were backcrossed 6 additional times to the C57BL/6 (B6) background and then c-IAP2^+/H570A^ were interbred to obtain c-IAP2^H570A/H570A^ mice. B6 mice bred in the CRC Vivarium (NIH) were used as controls for all experiments.

### Ethics Statement

All animal experimental procedures were approved by the Animal Care and Use Committee of the National Cancer Institute.

### LR-PCR and Real-Time PCR

The fragment spanning the recombination arm containing c-IAP2^H570A^ was amplified from tail DNA using buffer 3 from the Expand Long Template PCR System (Roche) and the c-IAP2 locus 5′ and c-IAP2 locus 3′ primers, digested with Spe1, and resolved by agarose gel electrophoresis. Total RNA was isolated from purified B cells using the Utraspec RNA isolation reagent (Biotecx laboratory) and reverse transcribed using Superscript II Reverse Transcriptase kit (Invitrogen) following the manufacturers' protocol. The amount of ferritin heavy chain, IκB, c-IAP2, GADD45β, Bcl-2, and hypoxanthine phosphoribosyltransferase (HPRT) mRNA was quantified using the respective primers, SYBR Green PCR Master Mix (Applied Biosystems), and the 7500 Real Time PCR System (Applied Biosystems). The values were normalized to HPRT and the percent increase relative to wild type was calculated by dividing the c-IAP2 knockin values by the wild type values.

### Cell Preparation, Surface Staining, and Purification

Bone marrow, thymus, spleen, lymph nodes (superficial cervical, axillary, brachial, inguinal, and mesenteric) and GALT were harvested from wild type and c-IAP2^H570A/H570A^ mice, disrupted by teasing, and total cell suspensions made by gently mashing the debris through 40 µM nylon mesh (BD Biosciences). The cells were counted and the distribution of lymphoid populations in each organ was determined by cell surface staining and flow cytometry. B and T cells were purified from spleen and lymph nodes from wild type and c-IAP2^H570A/H570A^ mice using B and T cell enrichment kits following the manufacturer's protocol. The purity was determined by cell surface staining and flow cytometry, and for all experiments, greater than 90%. In some experiments B cells and splenocytes were cultured in RPMI supplemented with 10% fetal calf serum, 100 U/ml penicillin, 100 µg/ml streptomycin, 2 mM L-glutamine, and 50 µM-β-mercaptoethanol. MEFs were prepared from day 13.5 embryos as described [66] and maintained in Dulbecco's Modified Eagle's medium supplemented with 10% fetal calf serum, 100 U/ml penicillin, 100 µg/ml streptomycin, 2 mM L-glutamine, and 50 µM-β-mercaptoethanol.

### Cell Death and Proliferation

For quantifying cell death, splenocytes (7.5×10^5^ cells/ml) were incubated in vitro, stained with fluorescently labeled anti-B220/CD45R and anti-TCRβ, and incubated with 7-amino-actinomycin D (7AAD; 1 µg/ml). Uptake of 7AAD by dying B (B220+) and T (TCRβ+) cells was quantified by flow cytometry. The percentage of viable cells was calculated by dividing B220^+^7AAD^−^ or TCRβ^+^7AAD^−^ by the total B220^+^ or TCRβ^+^ cells at each time point. For BAFF- and anti-CD40-induced survival, purified B cells were incubated at (7.5×10^5^ cells/ml) with the indicated concentrations of BAFF or agonistic anti-CD40 (100 ng/ml) for 66 h, stained with fluorescently labeled anti-B220 and 7AAD, and analyzed by flow cytometry. To assess proliferation, purified B cells (2.5×10^5^ cells/ml) were stimulated with anti-μ F(ab′)_2_ (Jackson ImmunoResearch Laboratories, Inc.) or LPS (Sigma), and during the final 18 h of the 66 h period, DNA synthesis was measured by adding 1 µCi ^3^H-thymidine to the culture. The cells were then harvested and lysed, and the DNA was transferred to a filtermat. The amount of incorporated ^3^H-thymidine was quantified using a scintillation counter.

### Immunoblotting and Transient Transfections

B cells, T cells, and MEFs were lysed in a buffer containing 20 mM Tris pH 7.5, 150 mM NaCl, 1% Triton X-100, 1 mM EDTA, 30 mM NaF, 2 mM sodium pyrophosphate supplemented with Complete (Roche) protease inhibitor cocktail, and the detergent-soluble lysate was collected after centrifugation. Lysates were normalized to protein concentration, denatured in sample buffer (50 mM Tris pH 6.8, 10% glycerol, 2% SDS, 2% β-mercaptoethanol, and 0.04% bromophenol blue), resolved by SDS-PAGE, and immunoblotted with the appropriate antibodies. For knockdown studies, 3.0×10^5^ MEFs were plated in 60 mm cell culture dishes and 16 h later transfected with 30 nM of Universal Lo GC content non-targeting or c-IAP2 Stealth iRNA siRNA using Lipofectamine RNAiMAX (Invitrogen) following the manufacturer's protocol. After 24 h the cells were washed twice with phosphate-buffered saline (PBS) and lysed. For ectopic expression studies, 293T cells were transfected with the indicated plasmids using Lipofectamine 2000 (Invitrogen) following the manufacturer's protocol. Twenty-four hours later the cells were harvested, washed with PBS, counted, and lysed in sample buffer.

### In Vitro Protein Binding

Glutathione S-transferase (GST)-tagged proteins were expressed in DH5α cells with 0.05 mM isopropyl-β-thiogalactopyranoside at 16°C for 20 h and lysed in 20 mM HEPES pH 7.5, 100 mM NaCl, 1.5 mM MgCl_2_, 1% Triton X-100. The recombinant proteins were purified from clarified lysates using glutathione Sepharose 4B beads (Amersham Biosciences). The beads were washed extensively and incubated with ^35^S-labeled TRAF2 that had been translated in vitro using the TNT Quick Coupled Transcription/Translation System (Promega) for 3 h at 4°C in binding buffer containing 120 mM NaCl, 10% glycerol, 1% Triton X-100, and 50 mM Tris pH 7.5. The bead-bound complexes were washed with the binding buffer, eluted with sample buffer, and resolved by SDS-PAGE.

### Adoptive Transfers

Equal number of splenic B cells purified from wild type (CD45.1^+^) and c-IAP2^H570A/H570A^ (CD45.2^+^) knockin mice were mixed and 10^7^ cells were injected into the tail veins of RAG2-deficient (CD45.2^+^) mice. Forty-five days later the percentage of wild type and c-IAP2^H570A/H570A^ B cells in the spleens and lymph nodes was determined by staining cell suspensions with B220, CD45.1, and CD45.2 and analyzed by flow cytometry. The ratio was generated by dividing the percentage of c-IAP2^H570A/H570A^ B cells by the percentage of wild type B cells.

### Histology and Serum Immunoglobulin Titers

Mice were euthanized using CO_2_ inhalation and necropsies were performed. A comprehensive set of organs and tissues were collected and fixed in 10% neutral buffered formalin. Tissues were paraffin-embedded, sectioned at 5 µm, and stained with hematoxylin and eosin. For lymphocytes, slides were stained with biotin-conjugated anti-B220/CD45R or anti-CD3. The antigens were retrieved by microwaving in EDTA (B220) or citrate buffer (CD3). Detection of B220 was performed using the avidin-biotinylated enzyme complex (Vector Laboratories) with 3,3′-diaminobenzidine (Sigma) as chromagen. Detection of CD3 was accomplished using the Rabbit Elite kit (Vector Laboratories) using 3,3′-diaminobenzidine as chromagen. Slides were counterstained with hematoxylin. Stained sections were evaluated by a boarded veterinary pathologist. Serum immunoglobulin isotypes were quantified by ELISA following the manufacturer's protocol. *p* values were calculated using GraphPad Prism and a two-tailed *t* test.

## Supporting Information

Figure S1 **Absence of c-IAP2 E3-activity activates the non-canonical NF-κB signaling pathway.**
(0.27 MB TIF)

Figure S2 **Targeting strategy for generating the c-IAP2^H570A/H570A^ mice.**
(0.19 MB TIF)

Figure S3 **c-IAP2/MALT1 fusion protein lacks E3 activity.**
(0.16 MB TIF)

Figure S4 **Lymphoid development and homeostasis in 7–12-wk-old c-IAP2^H570A/H570A^ mice.**
(0.60 MB TIF)

Figure S5 **T cell hyperplasia in GALT of wild type and c-IAP2^H570A/H570A^ mice.**
(4.39 MB TIF)

Figure S6 **Binding of in vitro translated and metabolically labeled TRAF2 to glutathione beads bound to recombinant GST-tagged murine c-IAP2 and c-IAP2^H570A^.**
(0.10 MB TIF)

## Abbreviations

7AAD: 7-amino-actinomycin D
BAFF: B cell activating factor
BIR: baculovirus IAP repeat
GALT: gut-associated lymphoid tissue
IKK: IκB kinase
LPS: lipopolysaccharide
LR-PCR: long-range polymerase chain reaction
MALT: mucosal associated lymphoid tissue
MAP: mitogen activated protein
MEF: murine embryonic fibroblast
TNF-R1: Tumor Necrosis Factor Receptor 1
TRAF2: TNF Receptor Associated Factor 2
