# Correction: Non-Canonical NF-κB Activation and Abnormal B Cell Accumulation in Mice Expressing Ubiquitin Protein Ligase-Inactive c-IAP2

**DOI:** 10.1371/journal.pbio.1002502

**Published:** 2016-06-23

**Authors:** Dietrich B. Conze, Yongge Zhao, Jonathan D. Ashwell

The authors would like to clarify the source of splice lines that can be detected in several figures where lanes from parts of the same gel were juxtaposed. To assure readers of the integrity of the data and demonstrate the source and relationship of the gel lanes, we present in S1 Raw Data the original unedited gel images, assembled by Dr. Conze, showing the original non-spliced gels for each affected panel: 1B, 1C, 1D, 7C, 7F, 7G, S1, and S3. In each panel, the lane numbers noted at the bottom of the published image correspond to the lanes used from the original gel image. The data have been verified by the *PLOS Biology* Editors and the splicing does not affect the authors’ conclusions.

## Supporting Information

S1 Raw Data Includes the original data for Figure 1 B-D, Figure 7 C, F,G, and Figures S1 and S3. (PDF)
