# Supplementary figures and images for: Non-Canonical NF-κB Activation and Abnormal B Cell Accumulation in Mice Expressing Ubiquitin Protein Ligase-Inactive c-IAP2
Source: PLoS Biol. 2010 Oct 26;8(10):e1000518. doi: 10.1371/journal.pbio.1000518 (PMC2964333; doi:10.1371/journal.pbio.1000518)

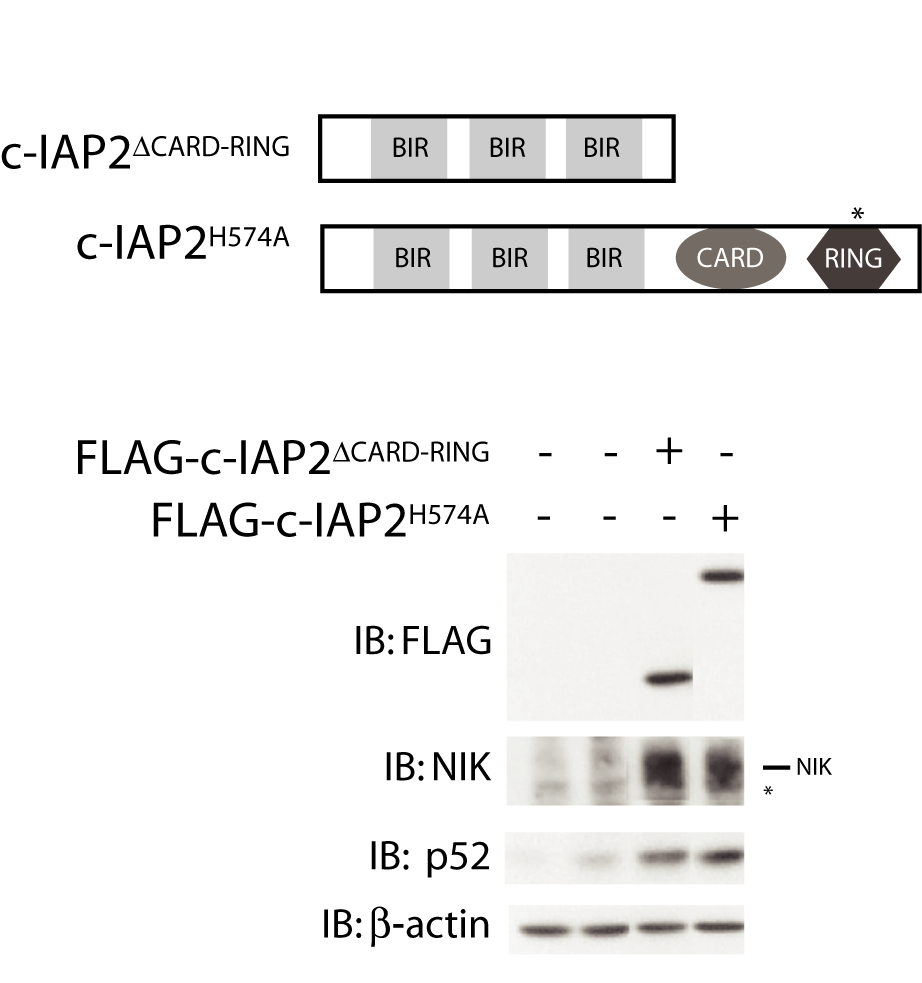

Supplement: Figure S1 — Absence of c-IAP2 E3-activity activates the non-canonical NF-κB signaling pathway. (0.27 MB TIF) [file pbio.1000518.s001.tif]

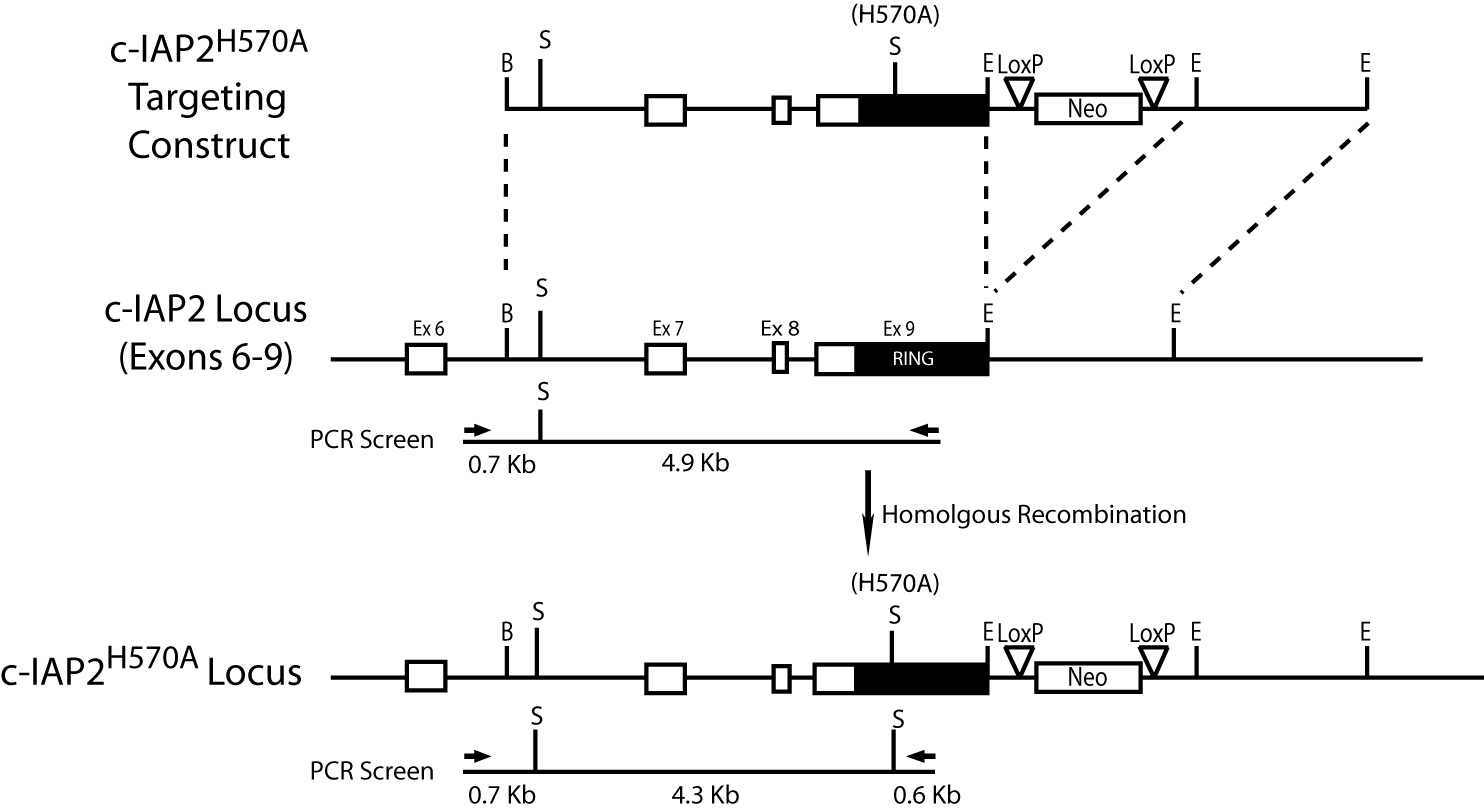

Supplement: Figure S2 — Targeting strategy for generating the c-IAP2H570A/H570A mice. (0.19 MB TIF) [file pbio.1000518.s002.tif]

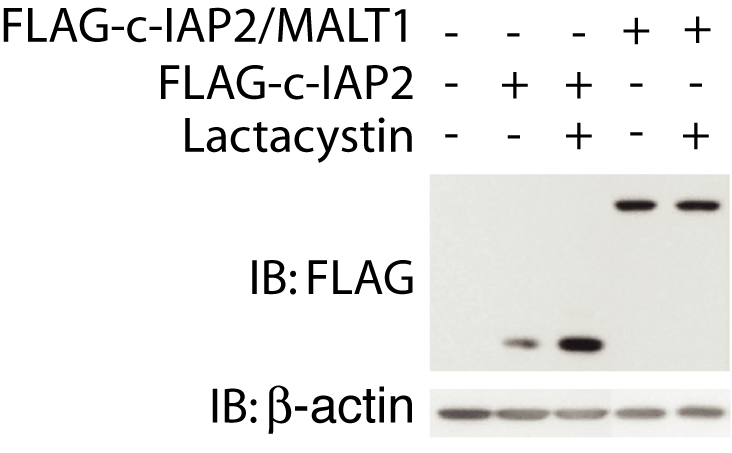

Supplement: Figure S3 — c-IAP2/MALT1 fusion protein lacks E3 activity. (0.16 MB TIF) [file pbio.1000518.s003.tif]

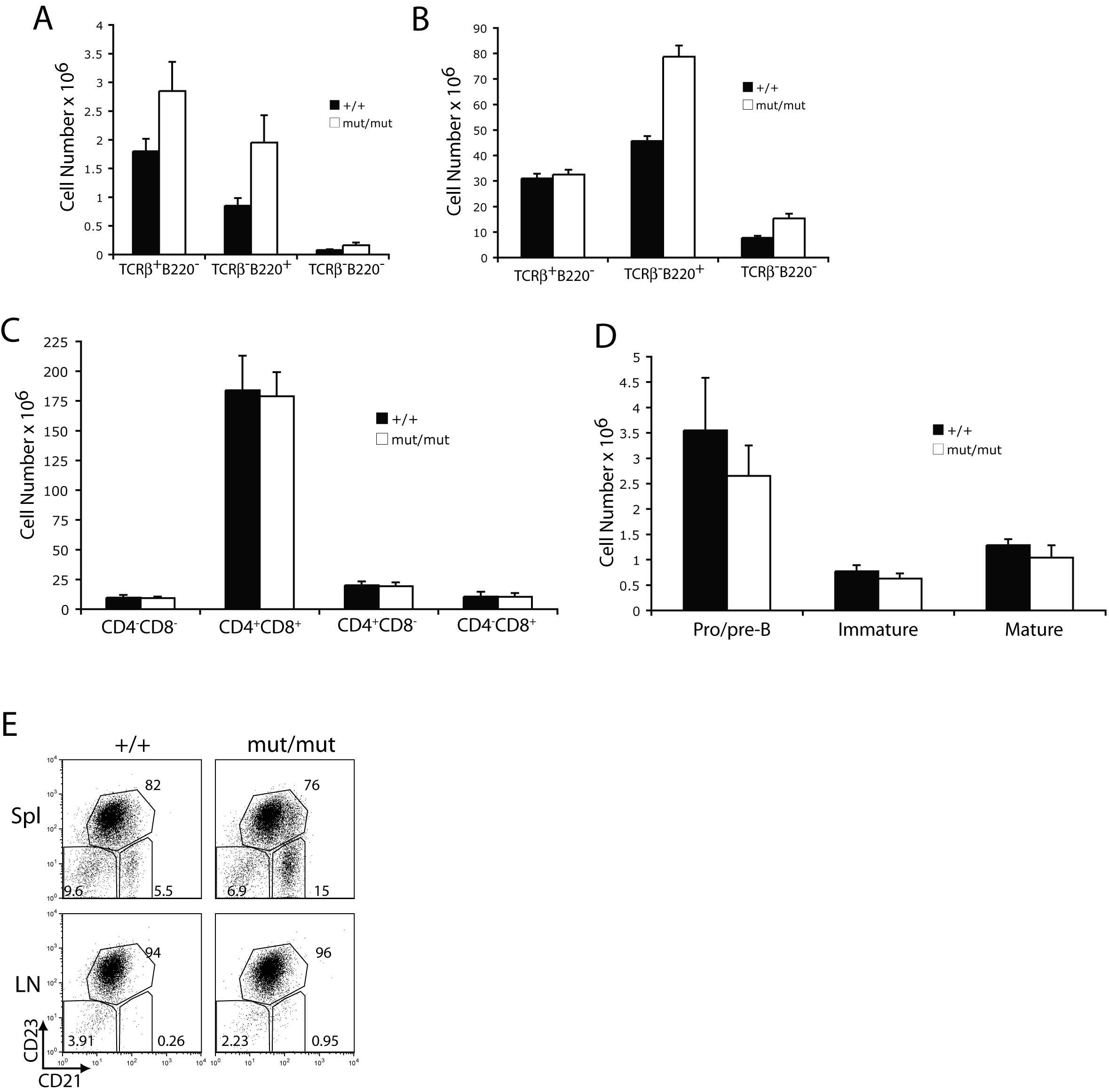

Supplement: Figure S4 — Lymphoid development and homeostasis in 7–12-wk-old c-IAP2H570A/H570A mice. (0.60 MB TIF) [file pbio.1000518.s004.tif]

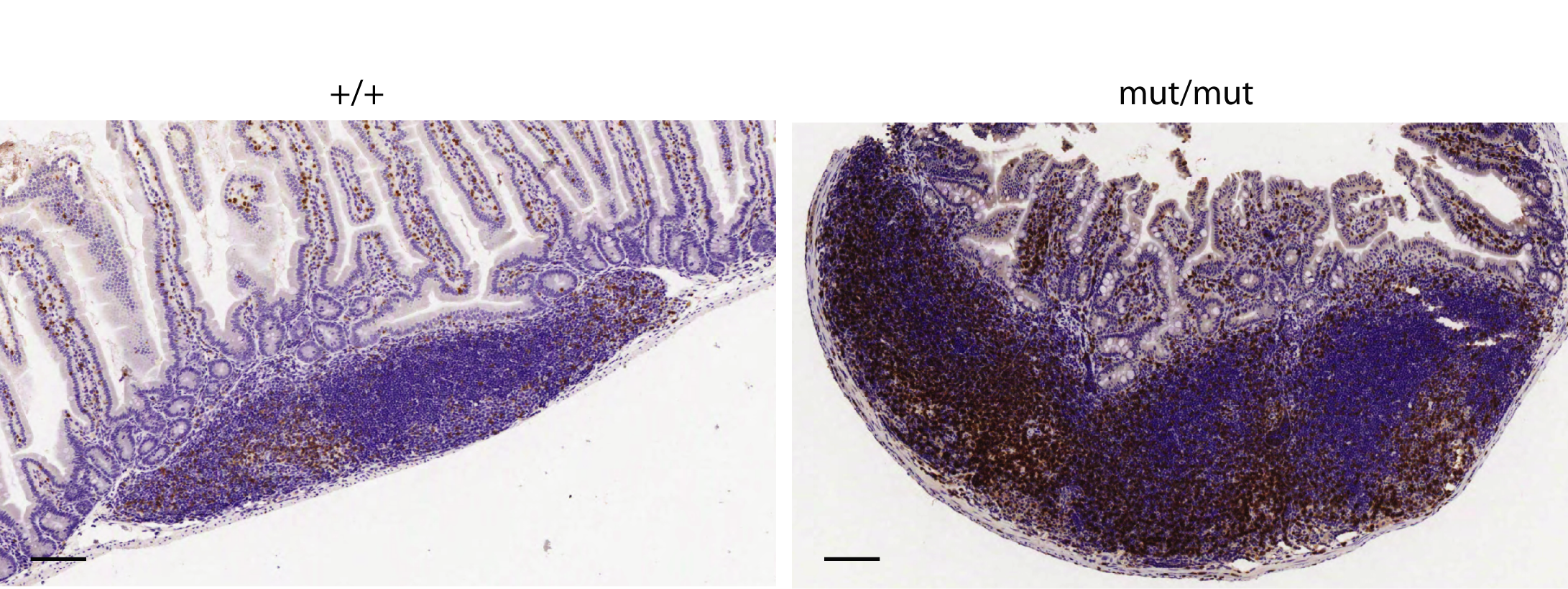

Supplement: Figure S5 — T cell hyperplasia in GALT of wild type and c-IAP2H570A/H570A mice. (4.39 MB TIF) [file pbio.1000518.s005.tif]

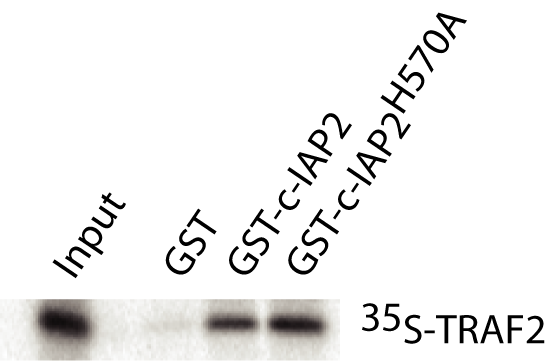

Supplement: Figure S6 — Binding of in vitro translated and metabolically labeled TRAF2 to glutathione beads bound to recombinant GST-tagged murine c-IAP2 and c-IAP2H570A. (0.10 MB TIF) [file pbio.1000518.s006.tif]
