# Supplementary material for: Correction: Non-Canonical NF-κB Activation and Abnormal B Cell Accumulation in Mice Expressing Ubiquitin Protein Ligase-Inactive c-IAP2
Source: PLoS Biol. 2016 Jun 23;14(6):e1002502. doi: 10.1371/journal.pbio.1002502 (PMC4919025; doi:10.1371/journal.pbio.1002502)

Figure 1B

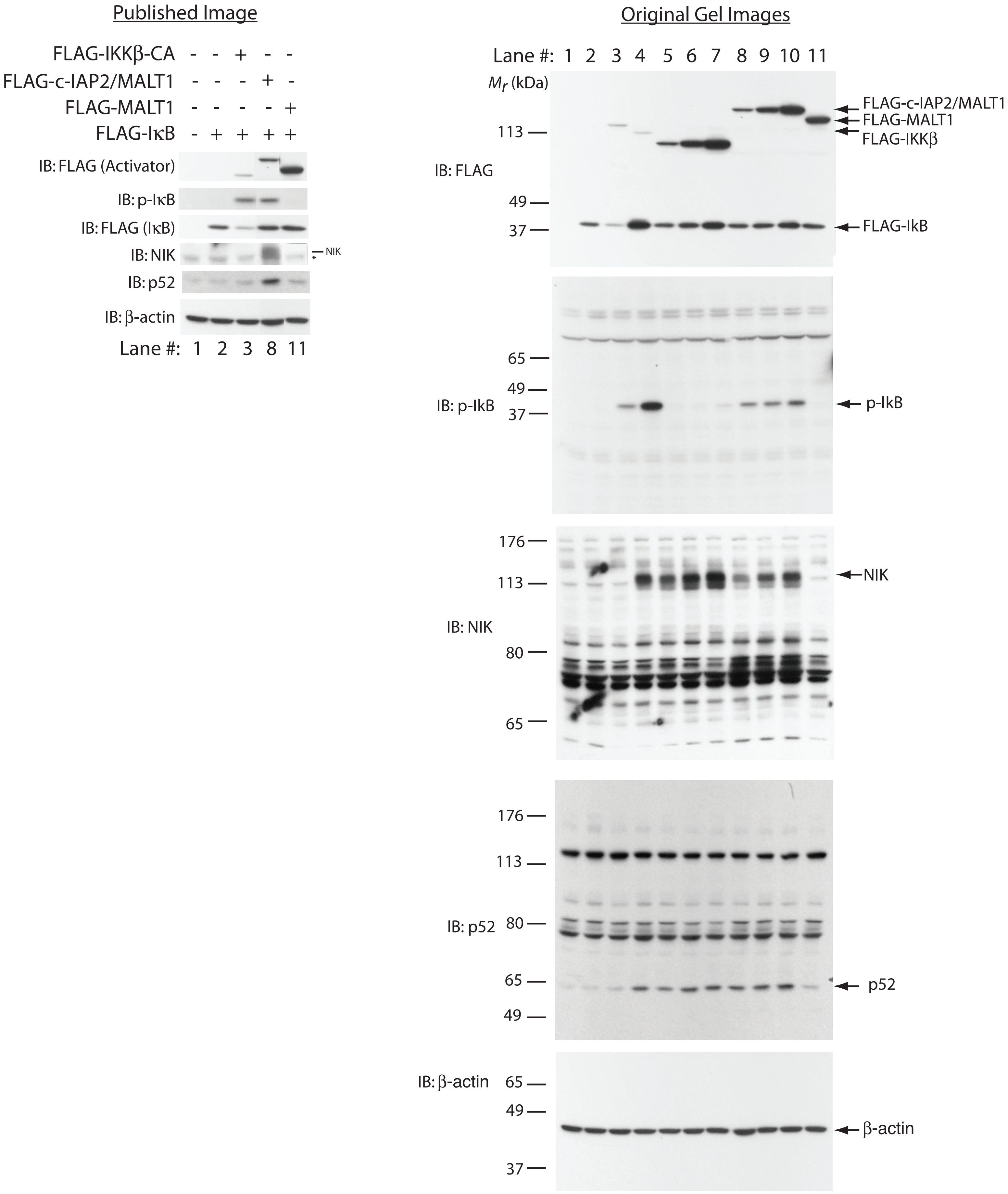
Published Image
FLAG-IKKβ-CA - - + - -
FLAG-c-IAP2/MALT1 - - - + -
FLAG-MALT1 - - - - +
FLAG-IκB - + + + +
IB: FLAG (Activator)
IB: p-IκB
IB: FLAG (IκB)
IB: NIK
NIK
*
IB: p52
IB: β-actin
Lane #: 1 2 3 8 11
Original Gel Images
Lane #: 1 2 3 4 5 6 7 8 9 10 11
Mr (kDa)
113
49
37
IB: FLAG
FLAG-c-IAP2/MALT1
FLAG-MALT1
FLAG-IKKβ
FLAG-IkB
65
49
37
IB: p-IkB
p-IkB
176
113
80
65
IB: NIK
NIK
176
113
80
65
49
IB: p52
p52
65
49
37
IB: β-actin
β-actin

Figure 1C

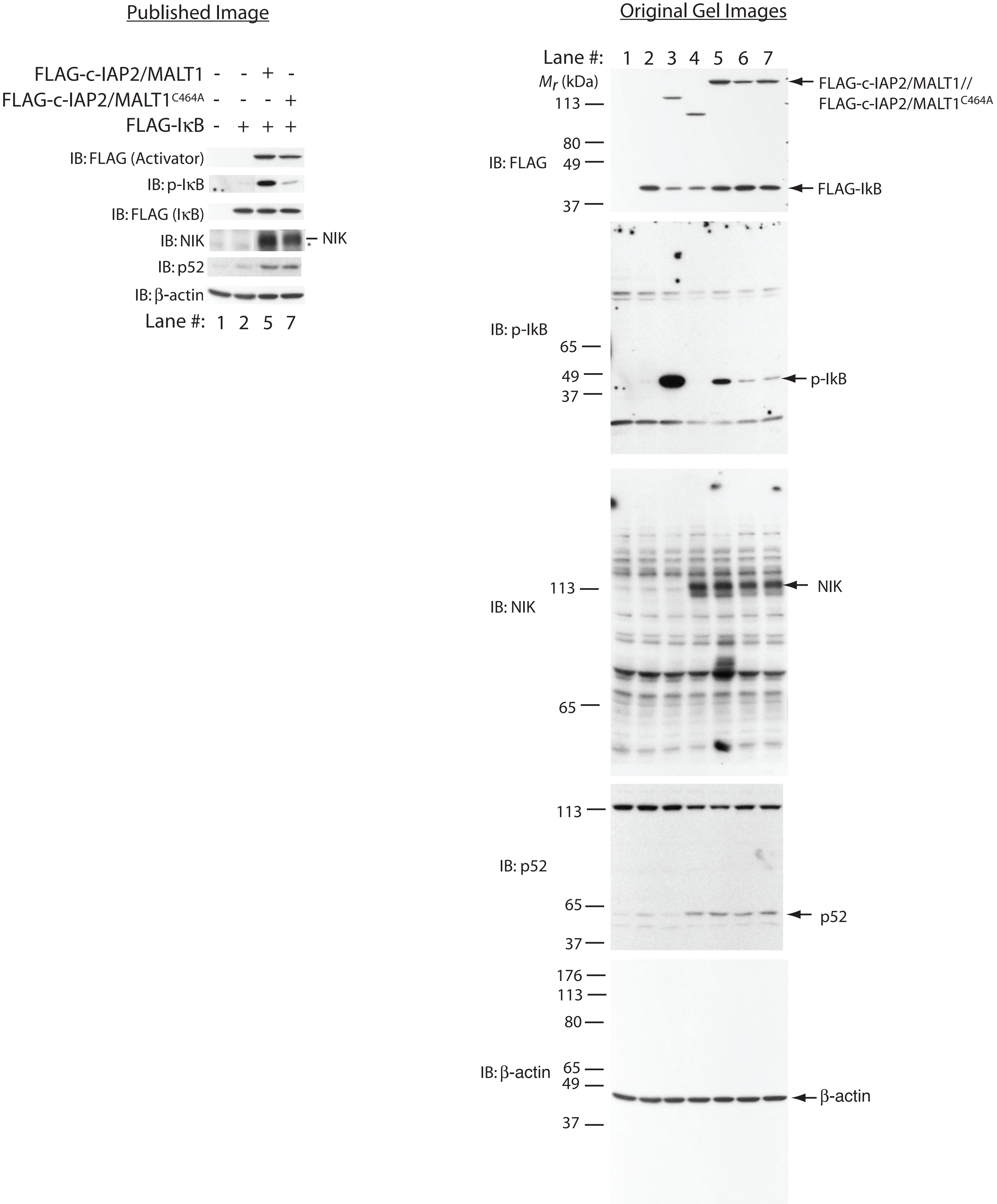
Published Image
FLAG-c-IAP2/MALT1 - - + -
FLAG-c-IAP2/MALT1C464A - - - +
FLAG-IκB - + + +
IB: FLAG (Activator)
IB: p-IκB
IB: FLAG (IκB)
IB: NIK
NIK
IB: p52
IB: β-actin
Lane #: 1 2 5 7
Original Gel Images
Lane #: 1 2 3 4 5 6 7
Mr (kDa)
113
80
49
37
IB: FLAG
FLAG-c-IAP2/MALT1//
FLAG-c-IAP2/MALT1C464A
FLAG-IkB
IB: p-IkB
65
49
37
p-IkB
IB: NIK
113
65
NIK
IB: p52
113
65
37
p52
IB: β-actin
176
113
80
65
49
37
β-actin

Figure 1D

Published Image

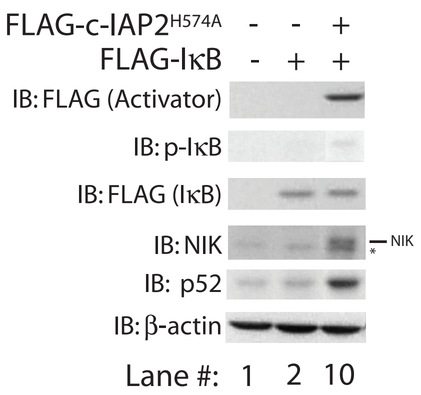

Original Gel Images

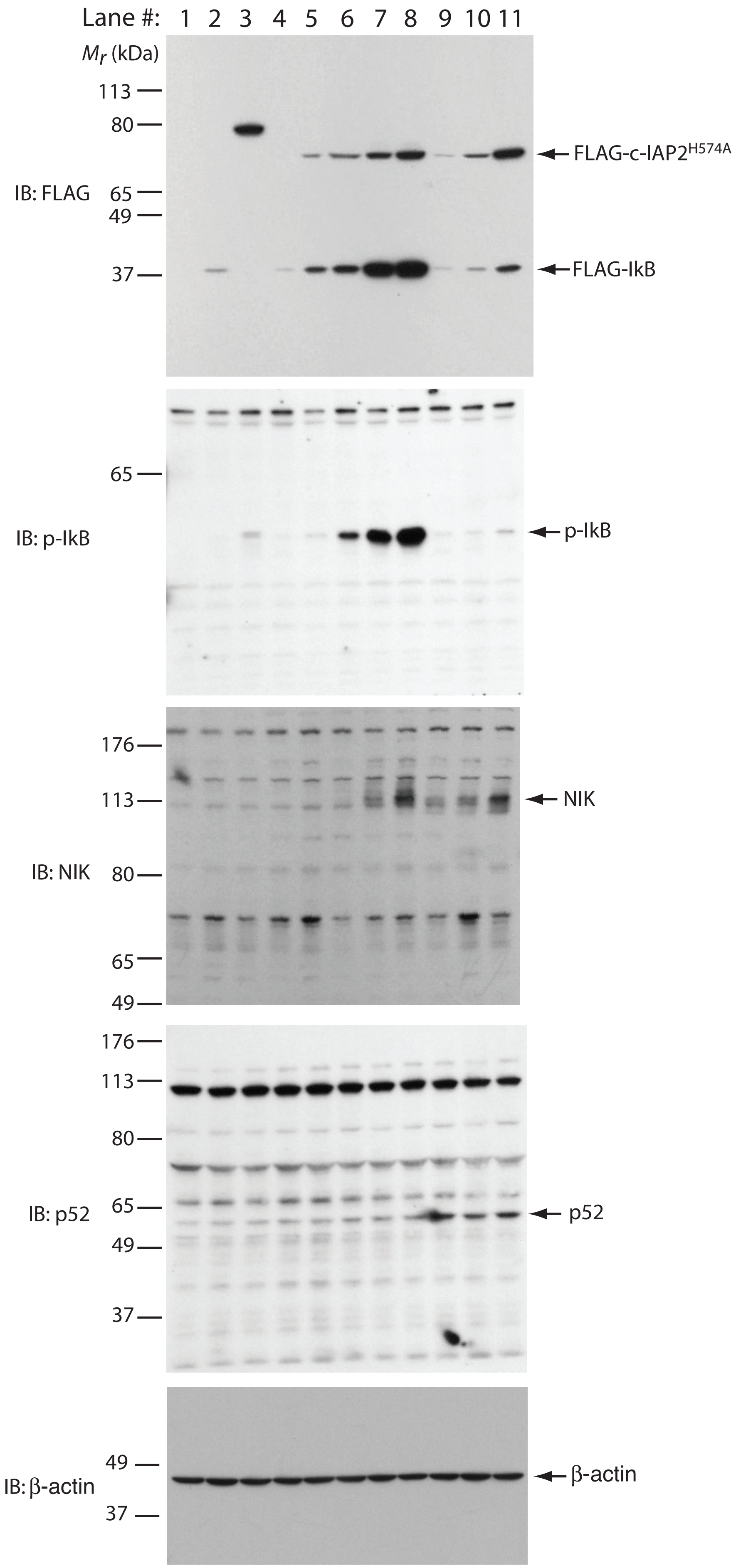

Figure 7C

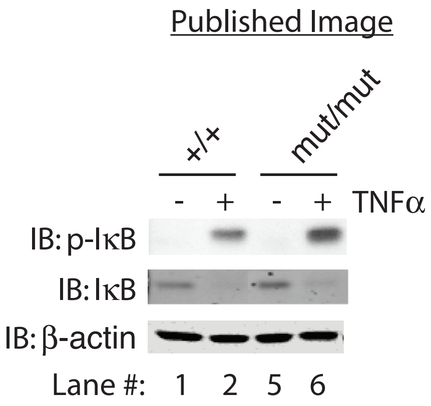

Published Image
+/+
mut/mut
- + - + TNFα
IB: p-IκB
IB: IκB
IB: β-actin
Lane #: 1 2 5 6

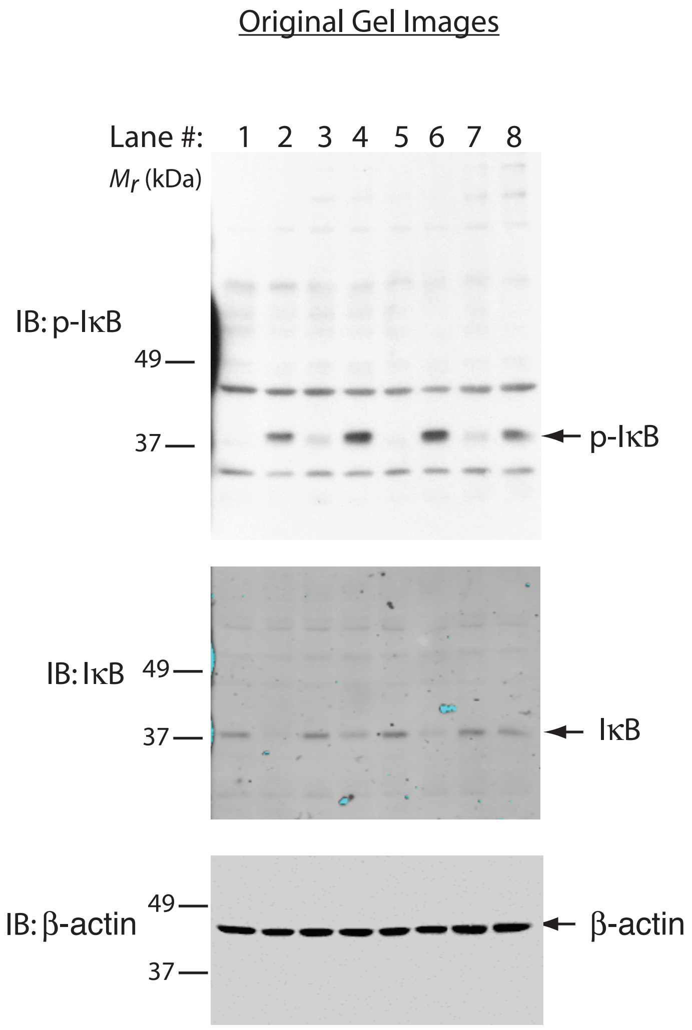

Original Gel Images
Lane #: 1 2 3 4 5 6 7 8
Mr (kDa)
IB: p-IκB
49
37
p-IκB
IB: IκB
49
37
IκB
IB: β-actin
49
37
β-actin

Figure 7F

Published Image

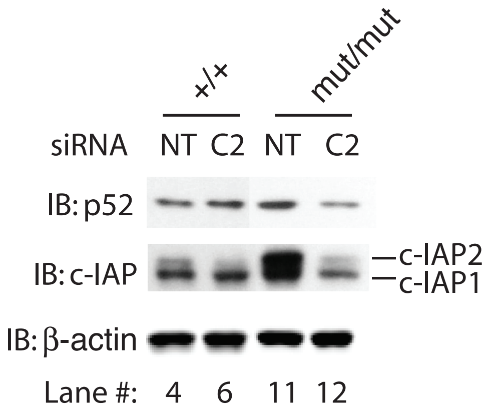

Original Gel Images

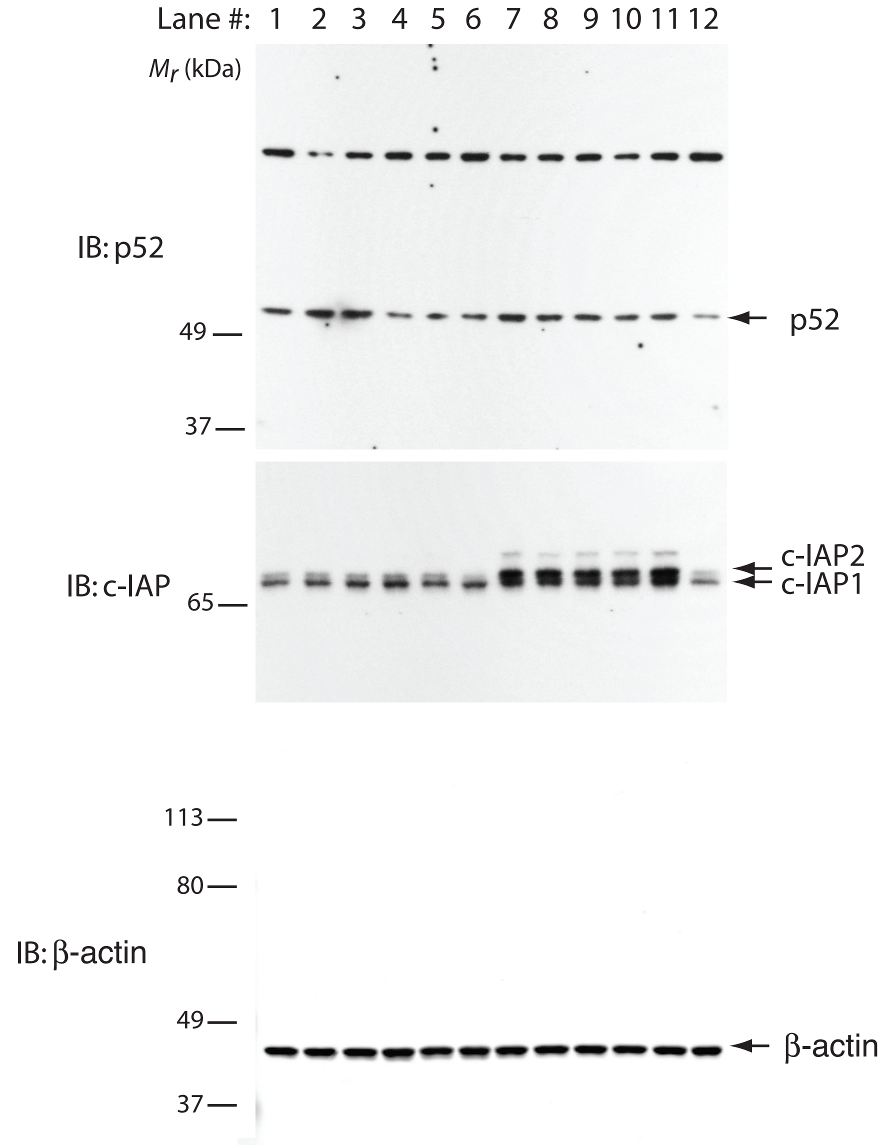

Figure 7G

Published Image

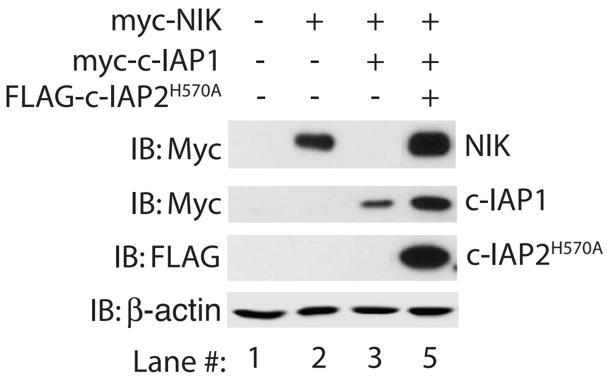

Original Gel Images

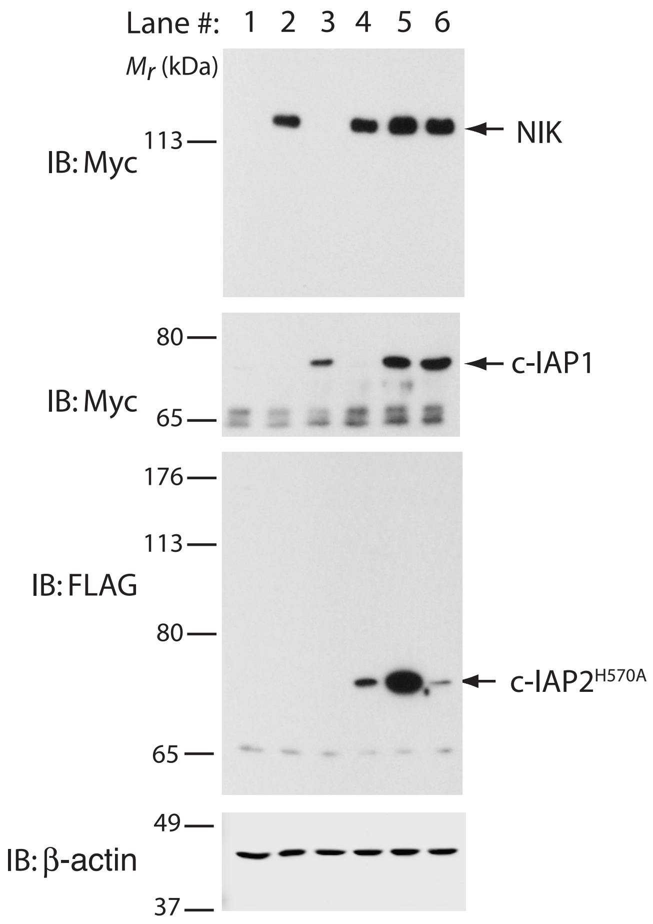

Supplemental Figure S1

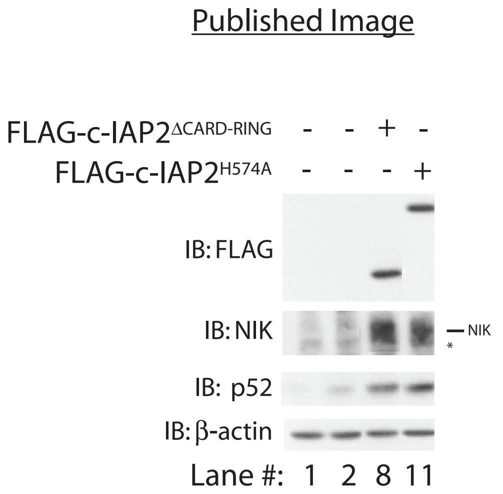

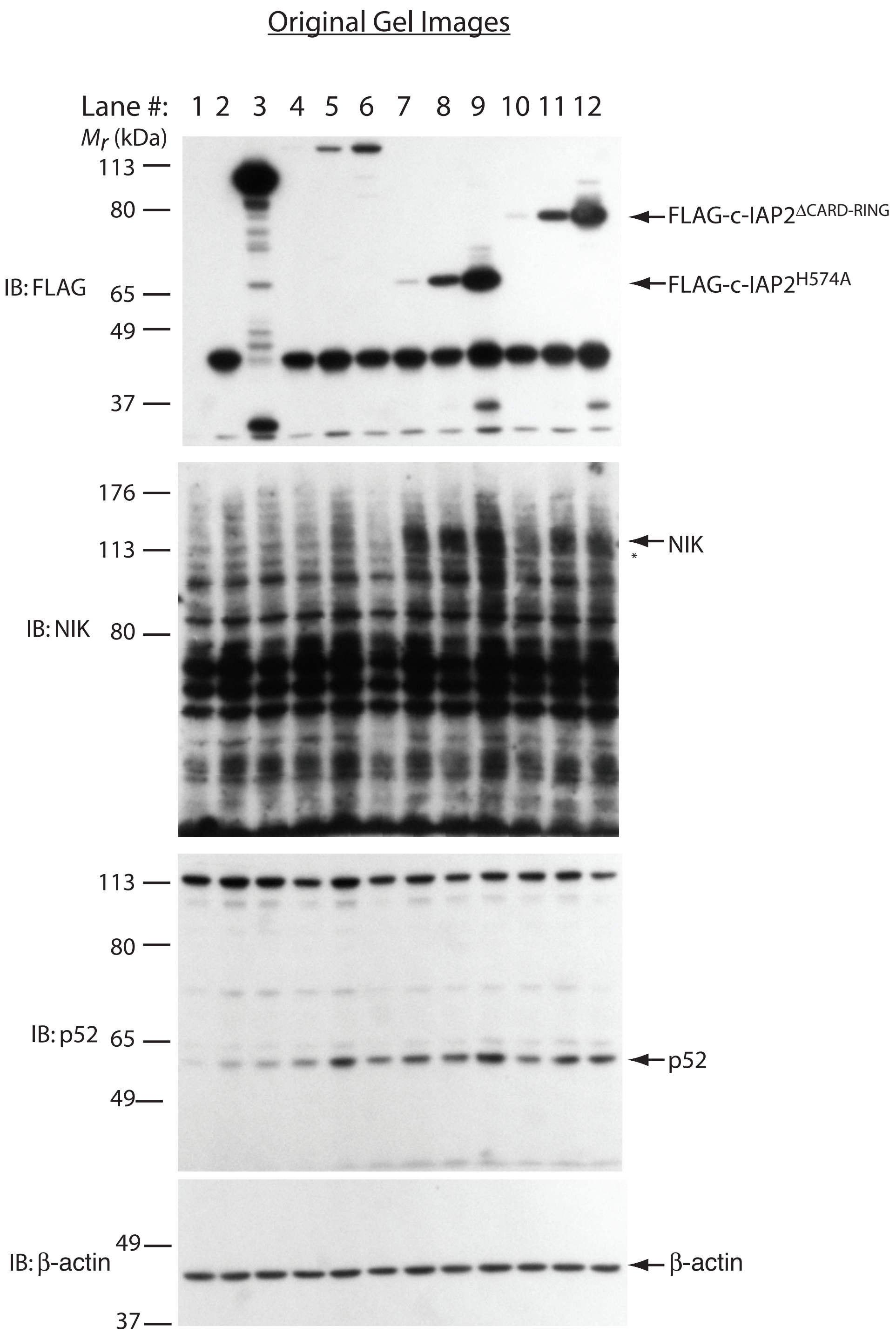

Supplemental Figure S3

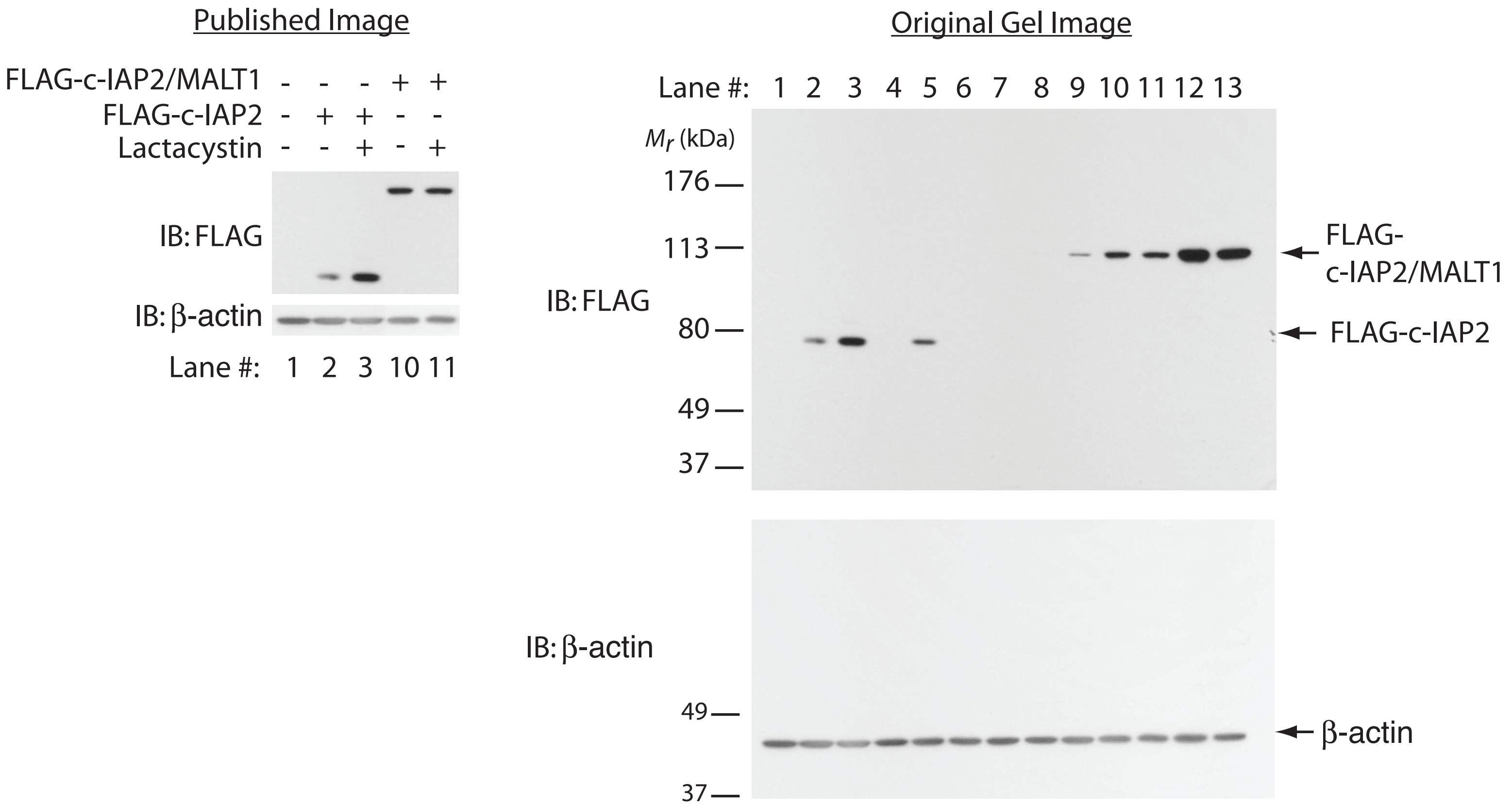

Supplement: S1 Raw Data — (PDF) [file pbio.1002502.s001.pdf]
